# Prosocial polio vaccination in Israel

**DOI:** 10.1073/pnas.1922746117

**Published:** 2020-05-26

**Authors:** Chad R. Wells, Amit Huppert, Meagan C. Fitzpatrick, Abhishek Pandey, Baruch Velan, Burton H. Singer, Chris T. Bauch, Alison P. Galvani

**Affiliations:** ^a^Center for Infectious Disease Modeling and Analysis, Yale School of Public Health, New Haven, CT 06520;; ^b^The Biostatistics & Biomathematics Unit, The Gertner Institute for Epidemiology and Health Policy Research, Sheba Medical Center, Tel Hashomer, 52621 Ramat Gan, Israel;; ^c^School of Public Health, The Sackler Faculty of Medicine, Tel-Aviv University, 69978 Tel Aviv, Israel;; ^d^Center for Vaccine Development and Global Health, University of Maryland School of Medicine, Baltimore, MD 21201;; ^e^Emerging Pathogens Institute, University of Florida, Gainesville, FL 32610;; ^f^Department of Applied Mathematics, University of Waterloo, Waterloo, ON N2L 3G1, Canada

**Keywords:** altruism, vaccination motives, disease prevention, game theory

## Abstract

Regions with insufficient vaccination have hindered worldwide poliomyelitis eradication, as they are vulnerable to sporadic outbreaks through reintroduction of the disease. Despite Israel’s having been declared polio-free in 1988, a routine sewage surveillance program detected polio in 2013. To curtail transmission, the Israel Ministry of Health launched a vaccine campaign to vaccinate children—who had only received the inactivated polio vaccine—with the oral polio vaccine (OPV). Determining the degree of prosocial motivation in vaccination behavior is challenging because vaccination typically provides direct benefits to the individual as well as indirect benefits to the community by curtailing transmission. However, the Israel OPV campaign provides a unique and excellent opportunity to quantify and model prosocial vaccination as its primary objective was to avert transmission. Using primary survey data and a game-theoretical model, we examine and quantify prosocial behavior during the OPV campaign. We found that the observed vaccination behavior in the Israeli OPV campaign is attributable to prosocial behavior and heterogeneous perceived risk of paralysis based on the individual’s comprehension of the prosocial nature of the campaign. We also found that the benefit of increasing comprehension of the prosocial nature of the campaign would be limited if even 24% of the population acts primarily from self-interest, as greater vaccination coverage provides no personal utility to them. Our results suggest that to improve coverage, communication efforts should also focus on alleviating perceived fears surrounding the vaccine.

## Significance

An individual’s decision to vaccinate can be motivated by both self-interest and prosociality, making it difficult to delineate the contribution of prosociality to vaccination uptake. A silent polio epidemic in Israel in which the primary purpose of vaccination was to avert transmission to the general community provides a unique case study through which we quantify, using game-theoretical models, the contribution of prosociality to vaccination decisions. We find that prosociality was a significant driver to rapidly achieving a high coverage of polio vaccination. To further boost coverage, public health communication efforts should be directed toward allaying fears about vaccine risks. Our approach is useful for enhancing participation in diverse disease control measures

Vaccination has achieved dramatic global reductions in polio disease burden. However, the final phases of polio eradication have been unexpectedly protracted (1–4) due to the persistence of cases in areas with variable vaccination coverage—Pakistan, Afghanistan, and Nigeria (5, 6). Insufficient vaccination in these regions has led to sporadic outbreaks and reemergence of the disease into countries where it has otherwise been eliminated (5–7).

Israel had been clear of wild poliovirus transmission since 1988, and in 2005 the country shifted to using the inactivated polio vaccine (IPV) (8). On 28 May 2013, wild poliovirus was detected in southern Israel after routine sewage sampling (7). Further surveillance of sewage and stool samples confirmed the spread of the virus and active transmission in humans (9). To curtail the outbreak, the Israel Ministry of Health proposed a campaign to vaccinate children under the age of 10 y with the oral polio vaccine (OPV) who had only received IPV (10). OPV elicits a relatively strong inhibitory humoral response that is essential for averting transmission and is advocated by the World Health Organization as the most effective approach for emergency outbreak control (11, 12). Since the initial IPV doses were sufficient to protect the individual against polio-associated morbidities, agreeing to receive OPV to prevent transmission entails a considerable degree of altruistic or prosocial behavior.

OPV is a live attenuated vaccine that can lead to vaccine-associated paralytic polio (VAPP) in one per million first doses, whereas IPV does not pose this risk (13). The risk of VAPP is circumvented by deploying a sequential schedule, initially immunizing with two IPV doses prior to OPV. This regimen protects recipients from the wild virus as well as VAPP, as was the case for the children targeted by the 2013 campaign. However, the reduced risk of VAPP associated with the Israeli schedule was not entirely comprehended by the public, as people tend to overestimate the likelihood of extremely rare events (14). This misperception of risk associated with VAPP highlights the prosocial nature of the campaign further, as individuals may believe they are putting themselves at potential harm in order to protect others.

The primary slogan to promote the 2013 OPV campaign in Israel had a strong prosocial context: “Just two drops, and the family is protected from the risk of polio” (15). Within the first few weeks of the campaign, 79% of the targeted population received a single dose of OPV (9). Prosocial behavior is an essential component for a spectrum of vaccination strategies, spanning from protection of one’s own child through maternal pertussis vaccination (16) to reducing future village-level malaria risk without guarantee of personal benefit via transmission-blocking vaccines (17). Previous work has shown that prosocial motivation is essential to understand vaccination coverage and that “pure” self-interested behavior will lead to lower-than-ideal vaccine uptake for disease control (18, 19). However, determining the degree of prosocial motivation is extremely challenging because the benefits from self-interest and from averting transmission in the community are usually intertwined (20). The particular circumstances of the vaccination program against the 2013 silent polio outbreak in Israel, where the social benefits were predominant, provides a prime case study to disentangle the self-interested and prosocial factors associated with vaccination decision making (7, 9, 21). A survey among Israeli parents examined the reasons for vaccinating their child with OPV during the prosocial vaccination campaign by focusing on self-interested and prosocial motives (*SI Appendix*, *Survey*) (22).

The conventional modeling approach for understanding vaccination behavior is through game theory. In classical game theory, vaccination is commonly conceived as an act of self-interest. Individuals decide either to protect themselves through vaccination or to “free ride” on the population-level protection built up by others (23, 24). When individuals are acting only in self-interest, the vaccination coverage required to obtain herd immunity might not be reached since the individual benefit of vaccination diminishes as vaccination coverage increases (23, 24). However, classical game theory can be augmented to include aspects of real-world decision making such as altruistic (prosocial) vaccination (18, 19) or bounded rationality (25).

We developed a game-theoretical model of a structured population that captures primary features of vaccination behavior among people in Israel during the 2013 silent polio outbreak. Using survey and epidemiological data from the Israel polio outbreak (Table 1), we parameterized our game-theoretical framework to address both prosocial motives and comprehension of prosocial nature of the vaccination campaign. We used the model to identify and quantify the drivers of vaccination coverage in a strategically interacting population where the prosocial vaccination program is occurring during a silent polio outbreak. We found that prosociality played a pivotal role in explaining the high observed vaccination coverage during the OPV campaign in Israel, which helped to offset fear regarding vaccine safety among parents.

**Table 1.** The degree of prosociality during the 2013 OPV vaccination campaign estimated from the game-theory model and results from the survey*

	National	Orthodox	Religious	Traditional	Secular	Arab
Vaccination coverage (ν)	72.1%	78.1%	63.6%	72.3%	63.7%	88.0%
Comprehended campaign as prosocial (α)	54.8%	48.4%	56.2%	63.6%	66.8%	25.5%
Estimated comprehensors that are prosocial (ρ)^†^	69.7%	74.5%	61.6%	71.1%	63.1%	80.2%
Minimum strength of prosocial behavior (κ)	0.24	0.27	0.21	0.24	0.21	0.32
Estimated strength of prosocial behavior (κ)^‡^	0.59	0.63	0.53	0.59	0.53	0.72
Sensitivity to infection (γ)	0.076	0.085	0.064	0.076	0.064	0.101

* We assumed a vaccine efficacy of 63%, 94% of the population is eligible for vaccination, the perceived basic reproductive number to be 2.24, the relative risk for an unaware individuals to be ∼2.14 × 10^−4^, the relative risk for an aware individual to be 0.001, and evaluating the probability of infection as *R*_0_(1 − εν)^1/γ^/(*R*_0_+(1 − εν)^1/γ^).

^†^ The percentage of the eligible population that is prosocial for the different sociocultural groups was estimated based on the assumption that unaware individuals were ∼1.22 times more likely to vaccinate than an aware individual (*SI Appendix*, *Validation*). Since 94% of the population was assumed eligible for vaccination (i.e., ω = 0.94), ωαρ represents the proportion of prosocials reported in the survey.

^‡^ Estimated using Eq. **4**.

## Results

We first constructed two alternative game-theoretic models to explain vaccination behavior during the 2013 Israeli silent polio outbreak: a “classical” individualistic model in which people act solely in self-interest and a prosocial model in which people can also be altruistically motivated to vaccinate in order to protect others. We found that the prosocial model is more consistent with both the vaccination coverage reported by the Israeli Ministry of Health and that estimated from the survey, irrespective of the population-level perception of wild-type polio risk (*SI Appendix*, Fig. S3). Specifically, the prosocial model was 17.4 times more likely than the individualistic model to achieve the 79% coverage reported by the Ministry of Health and 17.1 times more likely to achieve the 72% vaccination coverage reported by our survey (*SI Appendix*, Fig. S3).

### Data-Driven Population Stratification.

Survey data indicated that only 55% of the population comprehended the prosocial nature of the campaign, suggesting that a model with homogeneous behavior may not realistically represent the population. We therefore stratified the population into three groups based on their comprehension and prosociality (*SI Appendix*, Fig. S1). Specifically, if individuals understood the prosocial nature of the vaccination campaign, they were classified as aware, otherwise they were defined as unaware. Aware were further classified as prosocial or individualistic, where individualists were acting only to maximize payoff for their family (*SI Appendix*, Fig. S1). The proportion of the population in each category was parameterized by survey data (Table 1), such that 45% of the Israeli population were unaware, 17% were individualistic, and 38% were prosocial. The survey scores for questions regarding self-interested and prosocial motives were both high, suggesting that the two motives were important in the decision-making process (*SI Appendix*, Tables S3 and S4). Approximately 79% of people cited self-interest as the primary reason for vaccination. Among aware individuals, 67% cited fear of polio, child protection, or family protection as their primary reason. Therefore, the survey results suggest that individuals aware of the prosocial nature of the campaign still perceived a nonzero risk of morbidity from infection for themselves or their family. Therefore, to understand the vaccination uptake during the Israel OPV campaign, it is important to quantify the extent to which individuals accepted OPV vaccination because of self-interest and prosocial motivations or due to a misperception regarding their personal risk of paralysis.

For the prosocial population, the strength of prosocial behavior was estimated as 0.59, indicating that prosocials value the welfare of others at ∼59% of their personal welfare (*SI Appendix*, *Quantifying the Strength of Prosocial Behavior*). Weighted by the proportion of prosocial individuals, the average strength of prosocial behavior for the entire Israeli population is 0.23. Furthermore, analysis of the survey responses demonstrated that aware parents were more likely to perceive vaccine-related risk than parents who were unaware, and conversely unaware parents were more likely to perceive risk from wild virus (*SI Appendix*, Tables S6 and S7). In our model, we therefore introduced a term for the perceived relative risk of paralysis, quantifying the vaccine-related risk relative to the wild-virus risk. We estimated that the perceived relative risk of paralysis for an aware individual was ∼4.7 times greater than that for an unaware person (*SI Appendix*, *Risk and Perceived Risk*).

Perception of vaccine safety, which is directly proportional to the relative risk, was an important concern for 33.9% of the nonvaccinators, with 16.2% of the nonvaccinating parents stating it as their main reason for their decision (*SI Appendix*, Table S3). Concern about vaccine safety was different between aware and unaware parents, as it was given as the main reason for not vaccinating their child by 20% of aware parents and only 12% among unaware parents (*SI Appendix*, Table S7).

An increase in the relative risk can also be attributed to a decline in the perceived risk of wild-virus infection. Among parents who decided not to vaccinate their child, 45.2% understood that their child was already protected. This understanding of protection was the main reason for not vaccinating among 26.1% of nonvaccinating parents, stated in the survey by 27% of the aware parents and only 19% for the unaware parents (*SI Appendix*, Table S7). However, 85.7% of the parents who vaccinated their child fully agreed with the statement that they did so in order to protect their child (*SI Appendix*, Table S4). This expressed need to protect their child was lower among aware parents (53%) compared to unaware (69%) (*SI Appendix*, Table S6).

**Fig. 1.**
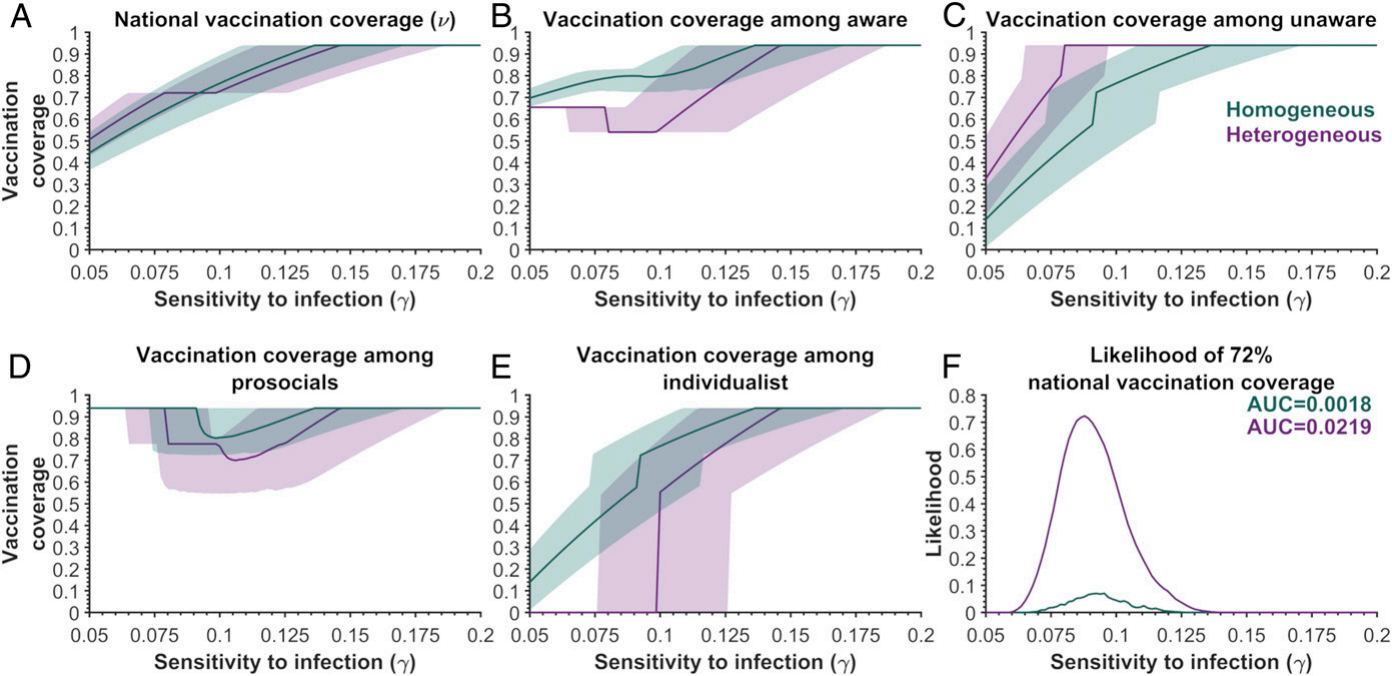
The vaccination coverages predicted by the Nash equilibrium for various sensitivities to infection (γ) among the aware and unaware groups for homogeneous relative perceived risk (green) and heterogeneous relative perceived risk (purple). (*A*) The predicted national vaccination coverage, (*B*) the vaccination coverage among aware individuals, (*C*) the vaccination coverage among unaware individuals, (*D*) the vaccination coverage among prosocials, and (*E*) the vaccination coverage among individualists. (*F*) The likelihood of achieving the national 72% vaccination coverage obtained in the survey. The results are based on 2,500 samples of relative risk of paralysis and *R*_0_ using the sigmoidal function. For the heterogeneous risk, the relative risk of the aware groups was assumed to be ∼4.7 times larger than the relative risk of the unaware group, having the same average perceived risk as the homogeneous population. The proportion of prosocial behavior in the aware population was 69.7% (ρ = 0.697) and the level of comprehension was 54.8% (α = 0.548). The vaccine efficacy was 63% (ε = 0.63), with 94% of the population being eligible for vaccination (ω = 0.94). We used the minimum strength of prosocial behavior (κ) in the prosocial population (Eq. **3**) for the sampled perceived relative risk and basic reproductive number, as well as the value of the sensitivity to infection. The area under the curve is denoted by AUC.

Incorporating differential risk perception between the aware and unaware increases the likelihood 12.4-fold that the 72% vaccination coverage (found in the survey) would have been achieved during Israel OPV campaign, compared to the scenario with homogeneous risk perception (Fig. 1*F*). If risk perceptions were similar, individualists and the unaware would have identical vaccination strategies, resulting in higher coverage for the aware population compared to the unaware population due to its prosocial members (Fig. 1 *C* and *E*). Thus, the model with heterogeneous perception of risk reproduces the higher observed vaccination coverage among the unaware compared to the aware population, which the homogenous model fails to do (Fig. 1 *B* and *C*), which can serve as an indication of the existence of heterogeneous risk perception.

### The Effects of Prosocial Comprehension on Vaccination Coverage.

**Fig. 2.**
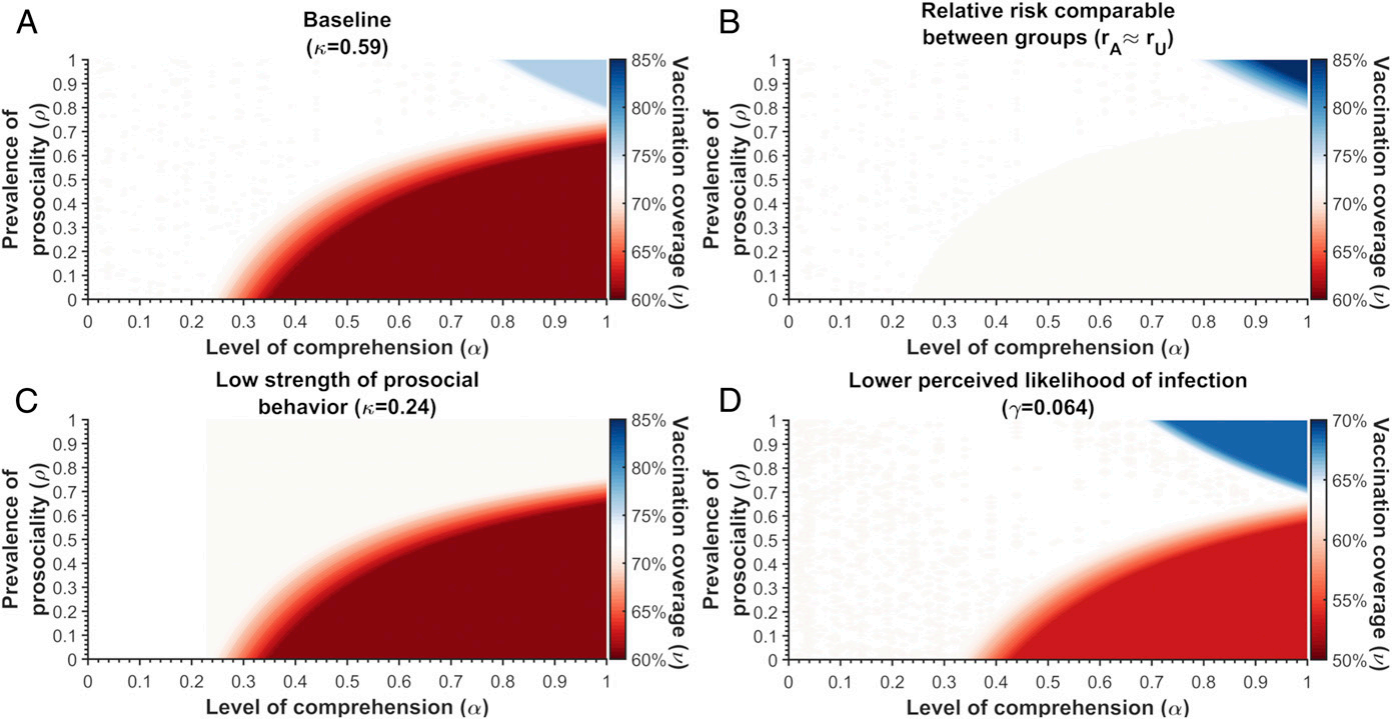
The vaccination coverage predicted by the Nash equilibrium across varying proportions of aware (α) and prosocial (ρ) individuals in Israel, given unaware individuals update their perceived relative risk. The blue denotes a coverage greater than baseline, red indicates a coverage lower than baseline, and white represents the baseline coverage of (*A*–*C*) 72% or (*D*) 64%. (*A*) The 72% vaccination coverage is based on the estimated κ = 0.59 and γ = 0.076 and the parameters values described in *SI Appendix*, Table S8. (*B*) The relative risk is relatively independent of awareness. We assumed that the perceived relative risk of the aware (*r*_*A*_) was 1% greater than the relative perceived risk for an unaware individual (*r*_*U*_). (*C*) The strength of prosocial behavior for prosocial individuals was assumed to be κ = 0.24 (i.e., the minimum estimate). (*D*) A reduced perceived likelihood of infection, where γ = 0.064 and the Nash equilibrium is 64%. The value of the perceived basic reproductive number, relative risk for the aware and unaware, and the fraction of the population eligible for vaccination are described in *SI Appendix*.

We next examined whether raising population-level comprehension regarding the prosocial nature of the campaign could have increased vaccination coverage. We calculated the Nash equilibrium coverage across a range of values for prosociality (ρ) and comprehension (α) (*SI Appendix*, *Solving Nash Equilibrium*). We first assumed that a currently unaware individual who becomes aware would also update their relative perceived risk perception to match that of a currently aware person, such that vaccination becomes comparatively riskier. Under this condition, increased comprehension would not generally result in higher vaccination coverage. If the proportion of prosocial individuals is less than 76.8%, increasing comprehension could lead to a drop in the vaccination coverage (Fig. 2*A*). This reduction occurs because individualists become less willing to vaccinate when they understand that there is no personal benefit (Fig. 1). For vaccination coverage to increase, high proportions of both prosocial behavior and comprehension would be required (Fig. 2*A*). The change in vaccination coverage is sensitive to the relative perceived risk of paralysis between aware and unaware individuals (Fig. 2*A* vs. Fig. 2*B*). If the perceived relative risk were comparable, increased comprehension would not reduce coverage, but high proportions of both prosocial behavior and comprehension would still be required for coverage to increase (Fig. 2*B*). If the strength of prosocial behavior among prosocial individuals (Eq. **3**) was at the lower-bound estimate of 0.24, no combinations of comprehension or prosociality could improve coverage (Fig. 2*A* vs. Fig. 2*C*). Finally, the combinations of comprehension and prosociality that result in an increase or decrease in vaccination coverage are dependent on the population’s original Nash equilibrium coverage, which is also an indicator of the perceived likelihood of wild-virus infection (Fig. 2*A* vs. Fig. 2*D*). For instance, if the original Nash equilibrium coverage had been 64%, equivalent to the lowest observed coverage in a subpopulation, then coverage could be improved even if only 67.7% of aware individuals were prosocial (Fig. 2*A* vs. Fig. 2*D*).

The effects of increasing comprehension are sensitive to the functional form chosen for the perceived likelihood of infection (*SI Appendix*, Fig. S4). We found that vaccination coverage was relatively stable across various values of prosociality and comprehension when the perceived likelihood of infection accounts for the full interruption of transmission at a given coverage (i.e., individuals “know” the herd-immunity threshold). Similarly, if individuals can “accurately” evaluate the final size of the outbreak, vaccination coverage would not change under any combination of comprehension and prosociality (*SI Appendix*, Fig. S4*D*). Given that individuals can accurately identify the herd immunity threshold, the estimated strength of prosocial behavior (κ) is estimated to be 44% lower compared to the base case at the national level (Table 1 and *SI Appendix*, Table S11).

We also evaluated the vaccination coverage for a scenario where currently unaware individuals become aware of the prosocial nature of the campaign but do not update their risk perception. In this case, increasing comprehension to 100% would only improve coverage if at least 85% of the unaware population is prosocial, because the newly updated unaware individualists become less likely to vaccinate as the prosocial population increases, as the high coverage within the prosocial population reduces the perceived risk for individualists (*SI Appendix*, Fig. S5).

### Prosociality by Subpopulation in the Model Reproduces Survey Results.

We examined whether the model developed for the general population of Israel would be able to predict subpopulation-specific survey results regarding the proportion of individuals who are prosocial. For each subpopulation identified by the survey—Orthodox Jews, Religious Jews, Secular Traditional Jews, Secular Jews, and Arabs—we found that the model with comprehension and prosociality can closely reproduce the proportion of each subpopulation that was prosocial (*SI Appendix*, Table S12 and *Validation*).

Across the Israeli subpopulations, the estimated strength of prosocial behavior (κ) ranged from 0.53 to 0.72 (Table 1), compared to the general population estimate of 0.59. Of note, the Arab population—where the outbreak was centralized—had the greatest strength of prosocial behavior among the five Israeli subpopulations (Table 1 and *SI Appendix*, Tables S9–S11).

## Discussion

In 2013, the Israeli Ministry of Health introduced an OPV campaign among IPV-vaccinated children to halt transmission of a silent polio outbreak (7, 9, 21). These circumstances provide a unique opportunity to study and quantify prosocial vaccination behavior, as the entire target population for the campaign was already protected from paralysis (7, 9, 21), and therefore had no “true” self-interest in vaccinating. By combining a game theoretical model with survey data, we identified partial population-level comprehension of the prosocial nature of the campaign and heterogeneous assessment of paralysis risks as crucial elements for reproducing the vaccination coverage observed during the Israel polio outbreak. Furthermore, we found that increasing comprehension would only improve coverage when both prosociality and comprehension of the prosocial nature of the campaign are very high.

During the 2013 Israeli OPV campaign, comprehension of its prosocial nature was associated with an increase in the perceived relative risk of paralysis (vaccine vs. infection) compared to those who did not understand the nature of the campaign. The incorporation of differential perceived risk between these groups was key for reproducing and understanding their observed differences in vaccine uptake. Without this heterogeneity in the perception of relative risk, one cannot achieve the observed heterogeneity in vaccination coverage. In addition to improving model realism, this discrepancy in perceived risk is reflected in the survey, which indicates that different information might have been available to each group. Specifically, 78% of the unaware parents vaccinated mainly to protect their child or from a fear of wild-virus polio, while only 59% of aware parents did so for these reasons. These results also highlight that neither group may have been acting with perfect knowledge about the campaign, as OPV does not provide their children with any additional protection against paralysis and has no risk of VAPP given previous vaccination with IPV.

Identifying the conditions where increasing comprehension in the population about the prosocial nature of the campaign could decrease vaccination coverage is critical to avoid undermining the public health objective. Our analysis revealed that the change in vaccination coverage is dependent on whether comprehension of the prosocial nature of the campaign is indeed connected to the perception of vaccine side effects and infection risks, the magnitude of that difference in risk perception between aware and unaware individuals, and how those perceptions change when a previously unaware individual becomes aware. We found that in a population with relatively homogenous perception of vaccine and wild-virus risks, there is no risk of coverage reduction even when comprehension increases within the population. An online experiment showed that communicating altruistic components of vaccination in a setting with low perceived risk improves the intention to vaccinate (26). Thus, the education about the vaccine to mitigate the effects of fears and misconceptions surrounding vaccine safety should be the primary focus of communication efforts, with altruistic elements being secondary.

As the polio outbreak in Israel entirely consisted of asymptomatic infections, it would have been difficult for most Israelis to assess whether and when vaccination had completely halted transmission. We therefore chose a function for the perceived likelihood of infection that assumes most individuals do not know the herd-immunity threshold. In our sensitivity analysis, we found that knowledge of this threshold can stabilize vaccination coverage across a much wider range of prosociality and comprehension, compared to the base case. For outbreaks in which infections are symptomatic, the general population may more accurately assess when the herd immunity threshold is reached and choosing one of these alternative functions might be more appropriate. In this case, it remains true that reducing the perceived risk of vaccine relative to wild virus is one of the few avenues for improving coverage. As with the base case, communication efforts should focus almost entirely on assuaging vaccine-related fears.

As expected, vaccination becomes more appealing to both individualist and unaware individuals at a higher perceived personal risk of infection. Similarly, a prosocial individual is more likely to vaccinate when the risk of paralysis to others is high, such that their personal vaccination can provide substantial marginal benefit. We evaluated the extent to which a prosocial individual values the health of others through the strength of prosocial behavior (κ). For this campaign, we estimated that the strength of prosocial behavior was highest in the Arab subpopulation, where the outbreak was centralized, but lower for other groups. This result is consistent with previous findings suggesting that prosocial behavior is often weaker when the benefit goes outside an individual’s own social group (27). For maximum impact, policymakers might consider concentrating prosocial campaigns within cohesive communities.

The survey data indicated that parents who decided to have their child vaccinated with OPV were responding to a combination of self-interested and prosocial motivations, with self-interest being the main reason given by most parents. This latter finding is somewhat surprising, given that vaccinated children derived no personal benefit from this campaign. Prosocial behavior has been examined previously in social sciences (28). However, evaluating the strength of prosocial behavior within a vaccination campaign is relatively new. The contribution of prosociality to influenza prevention has been quantified through survey studies (18, 29), but there have been no previous applications of a game-theoretical framework for quantifying prosocial behavior during a vaccination campaign. Furthermore, our study was uniquely able to connect the vaccination status for each survey participant with their responses. The influenza study estimate for the value that prosocial individuals assign to the health of others was 0.25 (18), which was consistent with the 0.23 population average calculated for our base case.

Although this OPV campaign represents the first pure prosocial vaccination program, there is a wide spectrum of altruistic behavior contributing to a variety of vaccination and disease control campaigns. The recent addition of boys to the human papillomavirus vaccine schedule was aimed at protecting their future female partners from cervical cancer. However, direct benefit to the boys is necessary to achieve high coverage, as only 24% of mothers would permit vaccination for this indirect goal (30). More routinely, healthy adults are vaccinated annually against influenza to reduce the infections among more vulnerable individuals, such as infants and the elderly (18, 29). Another example is from pertussis prevention, where parents and extended family are vaccinated to protect infants who have yet to obtain protective immunity (16, 31). If implemented, the use of a transmission-blocking vaccine against malaria would be a more “purely” altruistic vaccination campaign (17). The transmission-blocking vaccine targets the asexual stage of the malaria parasite, eliminating transmission to the mosquito, but does not offer the individual any direct protection from infection (17). Although this vaccine has not yet been used in the field, acceptance studies indicate that at least 71.8% would be willing to receive it (17). Our model structure is capable of analyzing the effects of prosocial behavior on vaccination coverage for these other diseases. Refining the understanding of prosocial vaccination within a population, and accounting for mixed self-interest and prosocial motives, complements past research and allows prosocial behavior to be harnessed further to improve vaccination coverage.

Prosocial behavior is a component of disease control measures beyond vaccination. Foremost examples are self-quarantine and self-isolation, critical behaviors for limiting the spread of newly emerged pathogens, such as SARS-CoV-2. Self-quarantine, performed by individuals who have been exposed to a confirmed case but not necessarily infected, could be considered a purely altruistic action. To prevent onward transmission, an individual forfeits sick days or faces a loss of income for 2 wk or until confirmation. Among the 80% of SARS-CoV-2 cases which are mild (32, 33), self-isolation is also a prosocial behavior. These mildly affected individuals could be well enough to attend school, work, or large public gatherings, but by isolating themselves in their home they are less likely to contribute to community transmission.

A classical paradigm for vaccination behavior states that self-interested agents will exploit herd immunity by not vaccinating, thereby reducing coverage below the thresholds necessary for disease control (25, 34) More recent research, including the present model, adds important insights and complexities to this paradigm (34). We have shown how the interaction between prosocial norms and risk perception can change outcomes in surprising ways. For example, it is superficially counterintuitive that increasing comprehension about the prosocial nature of the campaign can decrease vaccination uptake in an altruistic population. Only with these accompanying changes to risk perception do we observe this phenomenon.

Events in Israel following the silent polio outbreak may indicate that social norms about polio vaccination are changing. After the outbreak, the Israel Ministry of Health switched back to a routine vaccination schedule that includes two IPV doses followed by an OPV booster. This schedule has been well accepted among the Israeli population (35), possibly indicating a shift to a more prosocial norm. This shift could be driven both by a desire to increase indirect protection among contacts as well as stronger within-group sanctions against nonvaccinators (36). Alternatively, this shift in social norms could be attributed to a continuing lack of comprehension regarding the prosocial nature of receiving OPV. The work presented here thus provides a framework for assessing the recent evolution to a stronger prosocial norm for OPV vaccination in Israel.

Although a campaign might be designed as prosocial, the motives of the individuals and comprehension within the population both influence the ultimate vaccination coverage. Our analysis indicates that individualistic behavior can thwart the ability to avert transmission as comprehension of the prosocial nature of the campaign increases. These results could help guide the design of future OPV campaigns, in the drive toward polio eradication. As of April 2016, the World Health Organization initiated the switch from the trivalent OPV—poliovirus serotypes 1, 2, and 3—to a bivalent OPV—poliovirus serotypes 1 and 3 (37). Protection from serotype 2 will now be offered through IPV (38). As time passes, this policy change could allow for a silent outbreak, similar to Israel, through the reemergence of poliovirus 2. This type of reemergence is most likely in Pakistan, Afghanistan, and Nigeria, due to violence in the area’s limiting people from receiving the vaccine (39). In such an event, a monovalent OPV 2 campaign would be initiated, similar to the Israeli response in 2013 (10, 37, 38). Our analysis of the silent polio outbreak in Israel suggests that when promoting prosocial OPV campaign in an IPV vaccinated population, communication efforts should also focus on mitigating the fears and misconceptions surrounding vaccine safety.

## Methods

### Survey.

A total of 1,015 parents who had children aged 10 y and under were surveyed over the phone between January and June 2014 (22) regarding a vaccination campaign that began in August 2013, with a second campaign in early October 2013 (40). The study was approved by the Sheba Medical Center Helsinki Committee (Institutional Review Board approval no. 1826-14-SMC). Parents were informed that they were randomly chosen for a survey conducted for Gertner Institute for Epidemiology and Health Policy Research and all their answers would be kept confidential and only used for research purposes. All those contacted could opt out of the survey and could refuse to answer any question. The questionnaire focused on the parent’s comprehension of the prosocial nature of the campaign, motives for and against receiving OPV, and a risk assessment of the vaccine and wild-virus polio (*SI Appendix*, *Survey*).

### Game-Theoretical Model.

We developed an array of game-theoretical models (*SI Appendix*, *Game Theoretical Model*). The model which best fitted the data quantifies prosocial motives within a system where only some individuals comprehend the prosocial implications of a vaccination campaign. The polio outbreak in Israel consisted of asymptomatic infections, which would not allow for an individual to properly evaluate whether the vaccination coverage is sufficient enough to prevent all transmission (i.e., herd-immunity threshold). In addition, individuals cannot accurately evaluate their probability of becoming infected during an epidemic (18, 41, 42). Thus, our baseline perceived likelihood of infection, which builds upon a previously used form (43), does not account for the herd immunity threshold. We express the perceived probability of infection as a function of the basic reproductive number (*R*_0_), OPV vaccine efficacy (ε), OPV vaccination coverage (ν), and the sensitivity to infection (γ). The sensitivity to infection determines the rate at which the perceived probability of infection changes with respect to vaccination coverage (*SI Appendix*, *Utility Functions*). We denote the perceived probability of infection by Π(ν,ε).

The OPV vaccination coverage is dependent on the proportion of the target population eligible for OPV vaccination (ω), the percentage of the population that comprehended the prosocial nature of the campaign (α), and the fraction of those comprehending who are prosocial (ρ). Thus, the OPV vaccination coverage is expressed as

$$\nu =\omega \left[\alpha \left(1-\rho \right)q_{S}+\alpha \rho q_{P}+\left(1-\alpha \right)q_{U}\right],$$ [1]

where *q*_*S*_ is the probability of vaccination for an individualist, *q*_*P*_ is the probability of vaccination for a prosocial individual, and *q*_*U*_ is the probability of vaccination for an unaware individual (*SI Appendix*, *Utility Functions*). With a proportion of the population eligible for vaccination (i.e., ω), ωαρ represents the fraction of prosocials reported in the survey. Individualists are those who make vaccination choices based on their perceived risk of infection for themselves or their family, without consideration as to how their actions/inaction impact others.

The Nash equilibrium strategy is determined by the players’ payoff functions. A prosocial player’s expected payoff is

$$\begin{array}{l} E_{P}\left(q_{P},\nu ,\varepsilon \right)\equiv q_{P}\left[-r_{A}-\kappa \frac{\partial \Pi \left(\nu ,\varepsilon \right)}{\partial \nu }\left(1-\varepsilon \nu \right)-\left(1-\varepsilon \right)\Pi \left(\nu ,\varepsilon \right)\right]\\ \;\;\;\;\;+\left(1-q_{P}\right)\left\{-\Pi \left(\nu ,\varepsilon \right)\left[1+\kappa \left(1-\varepsilon \nu \right)R_{0}\right]\right\}, \end{array}$$ [2]

where *r*_*A*_ is the relative risk of perceived vaccine-associated paralysis risk vs. wild virus paralysis risk for a comprehending individual and κ is the relative amount a person values someone else’s health compared to their own, which we define as the strength of prosocial behavior (*SI Appendix*, *Utility Functions*). The marginal benefit of additional vaccination is −∂Π(ν, ε)/∂ν, which diminishes as more people become vaccinated. When not vaccinating, the prosocial individual incurs an additional burden due to potentially infecting others in the population (κ(1 − εν)*R*_0_). This burden is not accounted for in the payoff to vaccinate as the individual took action to prevent infection. We assumed that κ ≤ 1, indicating that individuals value their own health at least as much as they value that of others (28). When κ = 0, we obtain the expected payoff of an individualistic player (i.e., purely self-interested behavior) (*SI Appendix*, *Utility Functions*).

The survey indicates that perceptions of vaccination risk (i.e., VAPP) and infection risk likely differed between aware and unaware individuals (*SI Appendix*, Tables S6 and S7 and *Survey*). Thus, we allow for the unaware group, denoted *r*_*U*_, to have a lower relative perceived risk of paralysis than that of an aware person. An unaware individual does not consider the prosocial benefits of vaccination and cannot consider prosocial vaccination as a motivation for vaccination (*SI Appendix*, *Utility Functions*). Additional details on the derivation and motivation of the payoff functions for each type of player are in *SI Appendix* (*SI Appendix*, *Utility Functions*).

### Parameterization.

The proportion of the population who comprehended the prosocial nature of the campaign and the percentage of this population who were prosocial were informed by the survey results (*SI Appendix*, Tables S1 and S5). The efficacy of OPV and the percentage of children eligible to receive OPV were parameterized by previous estimates (7, 9, 10, 21, 44, 45). The perceived risk of VAPP vs. infection was informed by the disability weights and probabilities of the outcomes associated with the risk (*SI Appendix*, *Risk and Perceived Risk*). Using a maximum likelihood approach, we estimated the relative risk for an unaware individual and an aware individual (*SI Appendix*, *Risk and Perceived Risk*).

### Scenario and Sensitivity Analysis.

We compare a traditional individualistic model (ρ = 0) and a purely prosocial vaccination behavior (ρ = 1) using the likelihood of the observed vaccination coverage constructed from 2,500 samples of the perceived risk and *R*_0_ over range of the sensitivities of infection (γ). The area under the likelihood curve (AUC) is calculated to compare the two models, where a greater AUC implies a better explanation of the observed vaccination coverage. Using the same method, we then compared a model where the perceived relative risk is homogeneous (*r*_*U*_ = *r*_*A*_) to a model with heterogeneous perceived relative risk (*r*_*U*_ < *r*_*A*_).

To examine the effects of increasing comprehension of the prosocial motive of the campaign, we varied the proportions of aware (α) and prosocial (ρ) individuals. We conducted this analysis under the baseline scenario, comparable relative risk between the two groups, a lower quality of prosocial behavior, and a lower perceived likelihood of infection (i.e., a lower Nash equilibrium coverage).

#### Quantifying the strength of prosocial behavior.

The lower bound of the strength of prosocial behavior is estimated by

$$\kappa =\;\frac{r_{A}-\varepsilon \Pi \left(\nu ,\varepsilon \right)}{\left(1-\varepsilon \nu \right)\left[-\;\frac{\partial \Pi \left(\nu ,\varepsilon \right)}{\partial \nu }\;+R_{0}\Pi \left(\nu ,\varepsilon \right)\right]}\;,$$ [3]

where ν is the observed coverage from the survey (Table 1). We approximate the strength of prosocial behavior (κ) given the national vaccination coverage, without any knowledge of the prosocial equilibrium (Nash equilibrium in purely prosocial population), using

$$\kappa =\min\left\{\frac{\left[r_{A}+\left(1-\varepsilon \right)\Pi \left(\nu ,\varepsilon \right)\right]+\sqrt{{\left[r_{A}+\left(1-\varepsilon \right)\Pi \left(\nu ,\varepsilon \right)\right]}^{2}+4\left(1-\omega \right)\left(1-\varepsilon \;\nu \right){\left[\frac{\partial \Pi \left(\nu ,\varepsilon \right)}{\partial \nu }\right]}^{2}}}{-2\frac{\partial \Pi \left(\nu ,\varepsilon \right)}{\partial \nu }\left(1-\varepsilon \nu \right)},1\right\}.$$ [4]

We conduct a scenario and sensitivity analysis on the impact of our baseline values (*R*_0_, ε, ω, *r*_*U*_, *r*_*A*_, and α) on the model estimated values (*SI Appendix*, *Sensitivity Analysis*).

### Data Availability.

The summary of the survey results can be found in *SI Appendix*, *Survey*. The computational scripts used for the analysis can be found at https://github.com/WellsRC/Israel_OPV_2013.

## Supplementary Material

Supplementary File
