# Supplementary material for: Prosocial polio vaccination in Israel
Source: Proc Natl Acad Sci U S A. 2020 May 26;117(23):13138–44. doi: 10.1073/pnas.1922746117 (PMC7293608; doi:10.1073/pnas.1922746117)
Supplement: Supplementary File [file pnas.1922746117.sapp.pdf]

Supplementary Information:Prosocial polio vaccination in Israel

Chad R. Wells, Amit Huppert, Meagan C. Fitzpatrick, Abhishek Pandey, Baruch Velan, Burton H. Singer, Chris T. Bauch, and Alison P. Galvani

Survey

The survey about the oral polio vaccine (OPV) campaign was conducted between January and June 2014 over the phone and focused on parents in Israel who had a child age 10 and under during the outbreak (IRB Approval Number: 1826-14-SMC). A total of 1015 parents were surveyed about their opinions on the OPV campaign that the Ministry of Health initiated in August of 2013 [1]. The survey was for an internal report for the Israeli National Institute for Health Policy Research. The survey questions and results in the internal report are in Hebrew. We provide a brief summary of the survey results relevant to our paper and an English translation of the survey questions.

Summary of survey results

Using univariate analysis, we found that education level and income were statistically significant factors associated with vaccination. Specifically, higher levels of vaccination were observed in populations who were less educated, as well as in lower income families. We did not find any differences between people who declared that they vaccinated their children with: socioeconomic status, parent sex, age, number of children, or geographic area (i.e., South, Center or North).

A multivariate logistic regression found that education level and ethnic background are the two significant factors which explain the vaccine uptake. Non-academic vaccinated more than academic (OR = 0.7; 95% CI = 0.54–0.96; $P = 0.027$) and Arab vaccinated more than Jews (OR = 3.39; 95% Cl = 2.10–5.57; $P < 0.0001$). Within the Jewish population, high vaccine uptake was observed in very religious (Orthodox) and Traditional Jewish people (78% and 72% respectively). While lower vaccination uptake was observed in the Religious and Secular Jewish people (64%). In the multivariate analysis of the Jewish population, only religiousness was correlated with vaccine uptake. The Orthodox vaccinated more than the Secular (OR = 2.04; 95% Cl = 1.26–3.31; $P = 0.004$), and the Traditional also vaccinated more than the Secular (OR = 1.46; 95% CI = 1.014–2.097; $P = 0.042$). There were no differences between Religious and Secular Jewish people (OR = 0.98; 95% CI = 0.63–1.52; $P = 0.004$).

Main reasons for not vaccinating with OPV

The reasons for parents not vaccinating their child were divided into 11 categories

1. My child is vaccinated with inactivated polio vaccine (IPV) and is protected from infection
2. The vaccine is not safe enough and can cause side effects
3. There was no polio virus detected in the sewage next to my residential area
4. There were no cases of paralytic polio during the outbreak in Israel
5. I was not willing to vaccinate my child to protect other people
6. I did not believe the Ministry of Health
7. I do not believe in vaccination
8. I did not vaccinate because I was unable to make up my mind if I should vaccinate my child or not.

www.pnas.org/cgi/doi/10.1073/pnas.1922746117

9. I did not have time or interest in vaccinating my child.

10. The child could not be vaccinated due to other illness or an illness of a family member.

11. Other reason.

Parents were asked to grade each of the reasons above in their decision making process using a Likert scale (1-4, were 1 is no and 4 is very much). We also examine the number of parents in full agreement (i.e., the percentage of people which gave each reason the highest score) and the main reason behind the parent's decision not vaccinate their child (single answer). The reasons for not vaccinating where categorized into "self-interest" (reasons 1-4), negative attitude (reasons 5-7), passiveness (reasons 8-9), and different reasons (10-11). Self-interest was found to be the main factor which influenced parents not to vaccinate their children. Within the self-interest categories (1-4), the primary motive was the fact that the child had already been vaccinated with IPV, with concern about the safety of the vaccine being secondary. Of all the non-vaccinators, 49.8% choose a self-interest motive as their main reason for not vaccinating their children, while only 11% selected negative-attitude.

Main reason to vaccinate with OPV

The reasons for parents vaccinating their child were stratified into 10 categories

1. To protect my child
2. To protect my family
3. I was afraid of paralysis
4. Due to concern of public health in Israel
5. To protect other children at school
6. I trusted the Ministry of Health
7. We live in a country where polio can spread
8. In my family, the risk of polio is high
9. The doctor and/or nurse recommended to vaccinate
10. Other reason.

Similar to the case for reasons not to vaccinate, the parents had to grade the reasons for vaccinating their child using the Likert scale. We divided the reasons to vaccinate into "self-interest" (1-3), "social interest" (4-7) and other reasons (8-11). The primary reason for parents vaccinating their child was "self-interest", as all the scores for the three motives in this section where above 3.5. The pro-social vaccination reasons were deemed less important than self-interest, but also had scores over three. The Israeli public thought that to protect school children was an important component in their decision to vaccinate their children. We found that the public trust in the Ministry of Health (which is in charge of the public interest) was a major concern for parents when deciding to vaccinate their child. Thus, there is a balance between the self-interest and the public interest in the determining whether to vaccinate with OPV, as both reasons were indicated as key features in the decision process. However, 78.7% of parents indicated that self-interest was their main reason for vaccinating with OPV (a single reason), compared to the 11.2% indicating a pro-social main reason. The single major motive for vaccination identified among the parents was the need to protect their child (64.9%). Therefore, the main reason to vaccinate was self-interest with pro-social motives being an important and significant factor in the decision to vaccinate.

Parent's opinions regarding the vaccination campaign.

Parents who feared outbreaks (48.5%) were 1.3 more likely to vaccinate their children compared to those who did not. Parents who agreed with the claim that there was no need to vaccinate the children with OPV due to the fact that the children were already vaccinated (37.0%) were 1.6 times more likely not to vaccinate with OPV. People who thought the vaccine was given to stop transmission and not specifically to protect the vaccinated child (53.2%) were 1.2 less likely to vaccinate their child. Vaccination was twice as high in parents who thought that due to increased risk of an outbreak in the Middle-East there is a need to vaccinate with OPV (65.3%).

The dependency between parent's opinions and social demographic variables.

Lower vaccination rates were observed in the Jewish Secular and Religious populations (36.3% and 36.4% did not vaccinate respectively). Among the Traditional and Orthodox Jews there was moderate reduced vaccine uptake (27.7% and 21.9% did not vaccinate respectively). While in the Arab population only 12% did not vaccinate with OPV. In order to explain these observed patterns, we found that there were large differences in the amount of trust of the different groups to the Ministry of Health. While in the Secular population and in the Jewish Religious section the amount of distrust was high (35.1% and 32.2% respectively), in the Traditional Jews and in the Orthodox Jews it was moderate (26.7% and 18.5% respectively), and in the Arab population the level of distrust was low (13.6%). The kappa coefficient between vaccination and trust in the vaccination campaign was 0.472.

A second important correlation was found between vaccine uptake and risk perception. The risk perception was measured using two questions i) the need to vaccinate people who live in the Middle-East (a region with higher risk) ii) the need to vaccinate only the Bedouins (the core group of the outbreak). In the Secular population and in the Jewish Religious section, the risk perception was low and did not justify vaccination. The traditional Jews and in the Orthodox Jews had an intermediate risk perception and the Arab had the highest risk perception. The kappa coefficient was 0.396 and 0.391 for the regional and local risk respectively.

There was no significant kappa level between several other potential correlates such as: fear from polio, seasonal influenza vaccination, and there was no correlation between vaccine uptake and the different pro-social opinions between the different groups. Thus, there were differences between the above defined groups in there trust in the Ministry of Health and the risk perception which are correlated with vaccine uptake.

Comprehension of the pro-social aspects of the two drops vaccination campaign.

Comprehension was based on the level of agreement to the question "the live vaccine was mainly intended to stop the spread of the disease and not to protect the individual". Those who agreed with the statement (54.8%) were defined as aware. Unaware people were defined as those who did not agree with the statement (27.7%) or did not have an opinion (17.5%). Among the four Jewish populations 61.9% were aware, while only 25.5% of the Arab were considered aware. The differences between self-interest and pro-social interest are larger in the Jewish population compared to the Arab population. We have focused on the Jewish population to see if there are differences between comprehension and the social-demographic background. There was a strong increasing correlation between the comprehension and the following factors: education, income and more secular. There were no correlation between geographical location and age of the parent.

In the second stage, we divided the aware group into aware and vaccinated as well as aware and did not vaccinate. Among the aware who did not vaccinate, 185 individuals (22.5% of the Jewish population) claimed that they did not vaccinated due to the fact that there was no self-interest. Among the aware

parents who vaccinated their child, 324 (39.4% of the Jewish people) understood the pro-social aspect of the OPV campaign. The results from univariate model indicate that aware non-compliance is associated with: education, income and religiousness. In these people, comprehension seems to result in lower vaccine uptake. On the other hand there appears to be no correlation between aware compliance and the social demographic variables, emphasizing the fact that 40% of the Israeli population choose to behave in a pro-social manner seen in all levels of society.

Table S1: Vaccination coverage based perception of the oral polio vaccine (OPV) program

$p < 0.0001$	Opinion	Vaccinated (% N)	Not vaccinated (% N)
**There was no reason**	Do not agree ($N = 430$)	366 (85.12%)	64 (14.88%)
**to vaccinate with OPV since**	Agree ($N = 378$)	206 (54.50%)	172 (45.50%)
**children were already protected**	No opinion ($N = 207$)	160 (72.30%)	47 (22.70%)
$p < 0.0001$	Opinion	Vaccinated (% N)	Not vaccinated (% N)
**The program was aimed**	Do not agree ($N = 281$)	227 (80.78%)	54 (19.22%)
**at stopping the epidemic and**	Agree ($N = 556$)	364 (65.47%)	192 (34.53%)
**not protecting my child**	No opinion ($N = 178$)	141 (79.20%)	37 (20.80%)

Table S2: Responses to survey questions corresponding to socio-culture specification

**Motive**	**Behavior and Opinion**	**Orthodox** ($N = 128$)	**Religious Jews** ($N = 121$)	**Traditional Jews** ($N = 242$)	**Secular Jews** ($N = 322$)	**Arab** ($N = 184$)	**All** ($N = 1015$)
Compliance	Did not want to receive OPV	21.9%	36.4%	27.7 %	36.3%	12.0%	27.9%
Trust	Did not believe campaign was credible	18.5%	32.2%	26.7%	35.1%	13.6%	26.6%
Assessment of regional risk	Did not believe living in Middle East entails OPV vaccination	18.0%	32.3%	27.6%	38.8%	7.0%	26.5%
Assessment of local risk	Did not believe that universal vaccination was justified	24%	33.3%	26.3%	29.0%	11.4%	24.7%
Fear	Not afraid of epidemics	54.9%	60.3%	48.3%	64.6%	20.0%	50.5%
Program perception[a]	There was no reason to vaccinate with OPV since children were already protected	36.7%	36.4%	44.6%	40.0%	24.8%	37.5%
Program perception[a]	The program was aimed at stopping the epidemic and not protecting my child	66.0%	64.1%	74.4%	76.2%	34.3%	66.1%
Vaccination history	Did not vaccinate child against influenza	82.5%	71.7%	71.1%	71.9%	45.7%	68.4%

Table S3: The assessment of the motives for not receiving the oral polio vaccine among all respondents

**Motive**	**Reason for not vaccinating**	**Agreement score** (SN)	**Full agreement** (%)	**Main reason** (%)
Assessment	Understanding that your child is already protected	3.01 (1.18)	128 (45.2%)	74 (26.1%)
	Concerned about vaccine safety	2.84 (1.13)	96 (33.9%)	46 (16.2%)
	Polio was not detected in my community	2.20 (1.30)	69 (24.4%)	17 (6.0%)
	No reports of paralytic poliomyelitis	1.92 (1.14)	34 (12.0%)	4 (1.4%)
Negative sentiment	Not willing to vaccinate your child to protect others	2.14 (1.18)	54 (19.1%)	8 (2.8%)
	Not trusting the Ministry of health	2.05 (1.07)	36 (12.7%)	18 (6.4%)
	Being against vaccination	1.48 (0.87)	19 (6.7%)	5 (1.8%)
Inaction	Doubts about vaccination, finally did not vaccinate	2.13 (1.21)	51 (18.0%)	23 (8.1%)
	Was not interested in the subject	1.67 (1.01)	24 (8.5%)	7 (2.5%)
Different	Due to medical problems (child or family)	1.58 (1.10)	40 (14.1%)	34 (12.0%)
	Other reason			41 (14.5%)

Table S4: The assessment of the motives for receiving the oral polio vaccine among all respondents

**Motive**	**Reason for vaccinating**	**Agreement score** (SN)	**Full agreement** (%)	**Main reason** (%)
Self interest	Vaccinated in order to protect your child	3.76 (0.65)	627 (85.7%)	475 (64.9%)
	Vaccinated because of your fear from polio	3.58 (0.75)	509 (69.5%)	52 (7.1%)
	Vaccinated in order to protect your family	3.50 (0.91)	511 (69.8%)	49 (6.7%)
Prosocial interest	Vaccinated out of concern to the health of the public	3.06 (0.93)	260 (35.5%)	46 (6.3%)
	Vaccinated because you trusted the authorities	3.16 (0.87)	284 (38.8%)	24 (3 .3%)
	Vaccinated in order to protect other kids at school	3.06 (1.02)	297 (40.6%)	12 (1.6%)
Different	Middle East is a polio affected region	2.62 (1.14)	192 (26.2%)	11 (1.5%)
	Due to medical problems	2.12 (1.31)	192 (26.2%)	25 (3.4%)
	Pressure from HCV	1.32 (0.74)	27 (3.7%)	6 (0.8%)
	Other reason			25 (3.3%)

Table S5: Comprehension among the soci-cultural groups and corresponding vaccination uptake

**Socio-cultural group**	**Aware** (%)	**Aware and vaccinated** (%)	**Not aware and vaccinated** (%)
Orthodox ($N = 128$)	62 (48.4%)	48 (37.5%)	52 (40.6%)
Religious Jews ($N = 121$)	68 (56.2%)	40(33.1%)	37 (30.6%)
Traditional Jews ($N = 242$)	154 (63.6%)	104 (43.0%)	71 (29.3%)
Secular Jews ($N = 322$)	215 (66.8%)	125 (38.8%)	80 (24.8%)
Arab ($N = 184$)	47 (25.5%)	40 (21.7 %)	122 (66.3%)

Table S6: The main reasons parents decided to vaccinate their child stratified among those who were aware and unaware of the proscocial nature of the campaign

**Main reason**	**Aware**	**Unaware**
Vaccinated out of concern to the health of the public	12%	2%
Vaccinated because you trusted the authorities	4%	2%
Vaccinated because of your fear from polio	6%	9%
Vaccinated in order to protect your child	53%	69%
Pressure from HCV	1%	1%
Vaccinated in order to protect your family	8%	7%
State of disease	6%	2%
Vaccinated in order to protect other kids at school	2%	1%
Middle East is a polio affected region	2%	2%
Other	6%	5%

Table S7: The main reasons parents decided not to vaccinate their child stratified among those who were aware and unaware of the proscocial nature of the campaign

**Main reason**	**Aware**	**Unaware**
Being against vaccination	2%	1%
Not trusting the Ministry of Health	7%	3%
Was not interested in the subject	2%	3%
Understanding that your child is already protected	27%	19%
No occurrence	6%	5%
Concerned about vaccine safety	20%	12%
Not willing to vaccinate your child to protect others	4%	1%
Doubts about vaccination, finally did not vaccinate	7%	13%
State of disease	11%	14%
Other	14%	29%

Public's responsiveness to polio vaccinations – May 2014

Hello, my name is ___________________ and I am an interviewer from Tel Aviv University. I'm turning to you about research that we are conducting for the Gertner Institute for Epidemiology and Health Policy research, on the subject of vaccinating children against polio.
You have been chosen randomly to participate in this research project.
It is important that everyone who has been randomly chosen, participate in this research in order to ensure its quality.
We declare that your answers are kept confidential and will be used for research purposes only.
Thank you for your cooperation.

Filter question for interviewee:

Do you have children born between the years 2005-2012? [ages 1-10]

1. No – end of interview
2. Yes- continue

Interview parents to children aged 1-10

---

1. **How old are you?_____________**

For those who do not answer:

2. **To which age group do you belong?**

1.	18-24
2.	25-29
3.	30-34
4.	35-39
5.	40-44
6.	45-49
7.	50-54
8.	55-59
9.	60-64
10	65-69
11	+70
9.	Refusal to answer

3. **Sex:**

1. Male
2. Female

4. **What is your level of education?**
    1. Grade school
    2. Partial high school
    3. Full high school without Bagrut certificate (*Ed. Bagrut is a prerequisite for higher education in Israel*)
    4. Full high school with Bagrut certificate
    5. Higher education (teacher's seminar, nursing school, engineering college, yeshiva)
    6. Partial academic degree
    7. BA first degree
    8. Master's degree and above
    9. Do not read : refuses to answer

5. **In August 2013 Misrad habriut (The Ministry of Health (MOH)) began a national program called "Two drops" to immunize children with the live attenuated polio virus, administered by mouth. What was your immediate response when you heard that the MOH was beginning this immunization program?**

1. You knew immediately that you would immunize your children → go to question 8a
2. You knew instantly that you would <u>not</u> immunize your children → go to question 8b
3. You thought you should check further and think before deciding →continue
4. You did not relate to the subject initially → go to question 8
5. Do not read: you did not hear about the subject → go to question 9

If answered 3 to question 5:

6. **What did you do in order to come to a decision about whether to vaccinate your children against polio or not?**

May choose more than one answer:

.1 Followed the subject via media – TV, radio, newspaper
.2 Followed MOH explanations
.3 Consulted with health professionals (doctors, nurses)
.4 Consulted with family members and /or friends
.5 Read on internet
.6 Participated in discussions on the internet and social media, such as facebook
.7 Nothing special
.8 Other:
.9 Do not read: don't remember/ refuse to answer

7. **Did you manage to decide about whether to vaccinate your children against polio?**

1. You decided to vaccinate your children →go to question 8a
2. You decided not to vaccinate your children → go to question 8b
3. You didn't really decide, and continued to be indecisive → go to question 9
9. Do not read: don't remember/ refuse to answer

8a. From the following considerations, which most influenced your decision to vaccinate your child/ren during the "Two drops" national campaign to vaccinate against polio?
Mix 1-4

1. Your weighted decision after checking out the subject
2. Your belief and trust in the MOH
3. Your fear of polio
9. Do not read: don't remember/ refuse to answer
10. Do not read: other

8b. From the following considerations, which most influenced your decision NOT to vaccinate your child/ren during the "Two drops" national campaign to vaccinate against polio?
Mix 1-4

1. Your weighted decision after checking out the subject
2. Your disbelief and mistrust in the MOH
3. Your attitude towards vaccinations in general
9. Do not read: don't remember/ refuse to answer
10. Do not read: other

9. In the end, did you vaccinate your child against polio?

1. Yes
2. No→go to question 22

9a. Did you vaccinate:

1. Before the beginning of the school year
2. After the beginning of the school year

**How true is it that you vaccinated your child against polio because:**	**Not at all true**	**Not really true**	**Quite true**	**Very true**	**Do not read: don't know**
10. You were concerned about the health of the public	1	2	3	4	5
11. You trusted the MOH	1	2	3	4	5
11. Your fear of polio	1	2	3	4	5
12. To protect your child	1	2	3	4	5
13. Vaccinated due to pressure from doctor and/or nurse	1	2	3	4	5
14. In order to protect your family	1	2	3	4	5
15. Because an infant or elderly person reside in your house and you're concerned about their health	1	2	3	4	5
16. To protect other children at school	1	2	3	4	5

17. Because there is no alternative, as we live in a country where there may be polio outbreaks	1	2	3	4	5

20. Did you vaccinate your child against polio for another reason?
1. Yes, explain:
2. No

21. Which was the main reason that you vaccinated your child against polio?
1. Your concern about the health of the public
2. Your trust of the MOH
3. Your fear of polio
4. To protect your child
5. Pressure from doctor and/or nurse
6. To protect your family
7. Because an infant or elderly person reside in your house and you're concerned about their health
8. To protect other children at school
9. Because there is no alternative, as we live in a country where there may be polio outbursts

99. Do not read: don’t remember/ refuse

How true is it that you did NOT vaccinate your child against polio because:	Not at all true	Not really true	Quite true	Very true	Do not read: don't know
22. You don’t believe in vaccinations	1	2	3	4	5
23. You did not believe the MOH	1	2	3	4	5
24. You didn't get around to it or weren't interested	1	2	3	4	5
25. Your child has been vaccinated by inactivated vaccine and therefore is not at risk	1	2	3	4	5
26. There was no polio in the sewage in your area	1	2	3	4	5
26a There were no cases of polio countrywide	1	2	3	4	5
27. The vaccine is not completely safe and causes side effects	1	2	3	4	5
28. You're not prepared to vaccinate your child in order to protect others	1	2	3	4	5
29. Indecision	1	2	3	4	5
30. Due to someone being ill in your household	1	2	3	4	5

31. Did you NOT vaccinate your child against polio for another reason?
1. Yes, explain:
2. No

32. Among the reasons stated, which was your main reason for NOT vaccinating your child?
1. You don’t believe in vaccinations
2. You did not believe the MOH
3. You didn't get around to it or weren't interested
4. Your child has been vaccinated by inactivated vaccine and therefore is not at risk

5. There was no polio in the sewage in your area
5a There were not cases of polio countrywide
6. The vaccine is not completely safe and causes side effects
7. You're not prepared to vaccinate your child in order to protect others
8. Indecision
9. Due to someone being ill in your household
99. Do not read: don't remember/ refuse to answer

For everyone
33. Which of the following sentences best describes your family's decision whether to vaccinate your child/ren?
1. Mainly your decision
2. Mainly your partner's decision
3. Joint decision, totally in agreement
4. Joint decision, even though initially you were not in agreement
9. Do not read: don't remember/ refuse

34. To the best of your memory, what was your level of anxiety at the time of the discovery of the polio virus in the south:

High anxiety										Very low level of anxiety
10	9	8	7	6	5	4	3	2	1	0

To what degree do you agree or disagree to the contentions below about the "Two drops" program	Not at all true	Not really true	Quite true	Very true	Do not read: don't know
35. There was no reason to vaccinate with OPV since children had already been vaccinated with the inactivated vaccine	1	2	3	4	5
36. Polio was found mainly in Bedouin villages in the south, so no need to be vaccinated	1	2	3	4	5
37. The live virus was meant to prevent spread of disease and not to protect the vaccinated people.	1	2	3	4	5
38. In any case Israeli's are vaccinated with the live vaccine, due to high risk of polio in the Middle East.	1	2	3	4	5

39. MOH has a widespread campaign to convince the public to vaccinate children. Did you believe in the MOH campaign or not?
1. Not at all
2. Not so much

3. Believed somewhat
4. Believed fully

**To what extent does it anger you that…**		**Not at all true**	**Not really true**	**Quite true**	**Very true**	**Do not read: don't know**
39.	In a developed country like Israel there was a spread of polio in the sewage, and therefore you need to vaccinate your children	1	2	3	4	5
40.	The country expects you to vaccinate your children so that people who you do not know will not get polio infection.	1	2	3	4	5

To what extent does the following sentence describe you?

		Not so much	Quite true	True	Very true
41.	You have a terrible fear of plagues and will do all that is requested of you	1	2	3	4

43. To what extent does getting infected with polio frighten you compared to whooping cough?

1. Polio infection is far less frightening than whooping cough and measles
2. Polio infection is less frightening than whooping cough and measles
3. Polio infection is as frightening as whooping cough and measles
4. Polio infection is more frightening than whooping cough and measles
5. Polio infection is far more frightening than whooping cough and measles

44. In general, how concerned are you on a daily basis that something bad will happen to a family member

**Very concerned on a daily level**										**Not at all concerned on a daily level**
10	9	8	7	6	5	4	3	2	1	0

45. Did your child receive all the vaccinations recommended by the MOH in the first year of his life?

1. Received all the necessary vaccinations
2. Received some of the necessary vaccinations
3. Did not receive the necessary vaccinations

46. Did you vaccinate your children against flu last summer?

1. Yes
2. No

In conclusion, a few personal details for our statistics

47. At the time of the vaccination campaign, how many children under the age of 9 did you have?

48. At the time of the vaccination campaign, did you have an infant under the age of 1 year?
1. Yes
2. No

49. Family status
1. Live with partner
2. Live without partner
3. Do not read: refuses to answer

50. In which country were you born?

51. For those not born in Israel, year of immigration to Israel (4 digits)___________

52. Are you:
1. Jewish
2. Moslem
3. Christian
4. Druze
5. Do not read: refuses to answer

53. Jewish identity:
1. Very religious, haredi
2. Religious
3. Traditional religious
4. Traditional , not so religious
5. Not religious, secular
6. Not Jewish
7. Do not read: don't know/ refuse to answer

54. Arab identity:
1. Very religious
2. Religious
3. Not so religious
4. Not religious
5. Do not read: don't know/ refuse to answer

55. Today the average income in Israeli households is 14,000 shekels net.
Is your total income (both partners together) :
1. Much below the average

2. A little below the average
3. Average
4. A bit above the average
5. Much above the average
6. Do not read: not relevant- kibbutz member
7. Do not read: not relevant/ refuses to answer

56. Where do you live? (name of city/settlement)

<u>Thank you for your cooperation</u>

Game theoretical model

Utility functions

We denoted the vaccine efficacy of the oral polio vaccine (OPV) by ε and the vaccination coverage by ν. We assumed that each player plays a mixed strategy, where the player can either be an individualist (i.e. comprehends the nature of the campaign and self-interested), prosocial (i.e. comprehends the nature of the campaign and prosocial) or unaware (i.e. does not comprehend the nature of the campaign) (Figure S1). The proportion of the population eligible for OPV vaccination is denoted by ω. The proportion of the population that comprehends the prosocial nature of the campaign is denoted by α. We define an individual who comprehends the prosocial nature of the campaign as aware, and those who do not are termed unaware. The proportion of aware individuals who are prosocial is denoted by ρ. We denoted the probability of vaccinating for a prosocial individual by q_P, an individualist by q_S, and an unaware individual by q_U. Thus, we expressed the vaccination coverage as:

$$\nu = \omega \left(\alpha\rho q_P + \alpha(1-\rho)q_S + (1-\alpha)q_U\right). \tag{S1}$$

A measure of the risk of infection for an individual is required to form a payoff function for vaccinating and not vaccinating. However, individuals do not accurately evaluate their likelihood of becoming infected [2–4]. Thus,in our baseline analysis, we used the sigmoidal function

$$\Pi(\nu,\varepsilon) = \frac{R_0(1-\varepsilon\nu)^{1/\gamma}}{R_0 + (1-\varepsilon\nu)^{1/\gamma}} \tag{S2}$$

where $\gamma > 0$ is a constant that dictates the shape of the function (Figure S2) and represents the sensitivity to infection. We also considered three alternative functions to describe an individual's evaluation of their likelihood of becoming infected. One approach to estimating a probability of infection is to use a susceptible-infected-removed (SIR) epidemiological model:

$$\begin{aligned}
\frac{dS}{dt} &= -\beta SI \\
\frac{dI}{dt} &= \beta SI - \delta I \\
\frac{dR}{dt} &= \delta I
\end{aligned} \tag{S3}$$

where β is the transmission rate, and $1/\delta$ is the average duration of the infectious period, where $R_0 = \beta/\delta$ is the basic reproductive number. We expressed the estimated probability of infection as

$$\Pi(\nu,\varepsilon) = 1 - \frac{S_\infty}{1-\varepsilon\nu} = 1 - \frac{S_\infty}{(1-\omega-\varepsilon\nu)+\omega}, \tag{S4}$$

where $\frac{S_\infty}{1-\varepsilon\nu}$ is the proportion of the susceptible population not infected during the outbreak (Figure S2).To determine S_∞, one must first solve $\frac{dI}{dS}$ with respect to S and the initial conditions, and then set $I(\infty) = 0$. Thus, S_∞ satisfies

$$0 = -S_\infty + \frac{\ln(S_\infty)}{R_0} + (1-\varepsilon\nu) - \frac{\ln(1-\varepsilon\nu)}{R_0} \tag{S5}$$

and must be solved numerically [5]. The second alternative functional form is

$$\Pi(\nu,\varepsilon) = I_{max}(\nu,\varepsilon,R_0(1+\gamma)), \tag{S6}$$

where $\gamma > -1$ is a parameter constant to adjust the probability of infection (Figure S2) and the maximum prevalence of the SIR model is

$$I_{max}(\nu,\varepsilon,R_0) = \frac{\ln(1/R_0) - 1 + R_0(1-\varepsilon\nu) - \ln(1-\varepsilon\nu)}{R_0}, \tag{S7}$$

which is an approximation derived from the SIR system of equations (S3) [6] The third functional form we consider is a polynomial function

$$\Pi(\nu, \varepsilon) = I_{max}(0, 0, R_0)\left(1 - \varepsilon\nu\right)^{1/\gamma}, \tag{S8}$$

where γ is a parameter constant (Figure S2).

For the individualist, the payoff not to vaccinate is

$$-\,\Pi(\nu, \varepsilon) r_I^A \tag{S9}$$

which is just their perceived risk of becoming infected along with the risk of paralysis due to infection for an aware individual (r_I^A).

If an individualist decides to vaccinate,they incur risk of paralysis (r_V^A) due to side effects from the OPV as well as risk of infection if the vaccine is ineffective $\left(\left((1-\varepsilon) r_I^A \Pi(\nu, \varepsilon)\right)\right)$. Therefore, the payoff to vaccinate for an individualist is

$$-\,r_V^A - (1-\varepsilon) r_I^A \Pi(\nu, \varepsilon), \tag{S10}$$

and their expected payoff is

$$\begin{aligned} E_S(q_S, \nu, \varepsilon) &= q_S\left[-r_V^A - (1-\varepsilon) r_I^A \Pi(\nu, \varepsilon)\right] + (1-q_S)\left[-\Pi(\nu, \varepsilon) r_I^A\right] \\ &= -q_S r_V^A - \Pi(\nu, \varepsilon) r_I^A (1 - \varepsilon q_S). \end{aligned} \tag{S11}$$

We can factor out r_V^A such that

$$E_S(q_S, \nu, \varepsilon) \equiv -q_S r_A - (1 - q_S\varepsilon)\Pi(\nu, \varepsilon), \tag{S12}$$

where $r_A = r_V^A / r_I^A$ is the perceived relative risk of paralysis.

The payoff not to vaccinate for a prosocial is

$$-\,\Pi(\nu, \varepsilon)\left[r_I^A + r_I \bar{\kappa}(1 - \varepsilon\nu) R_0\right] \tag{S13}$$

where r_I is the perceived risk of paralysis due to infection for the population and $\bar{\kappa} \geq 0$ denotes the relative weight of the prosocial motive in the decision to vaccinate. For example, as $\bar{\kappa}$ increases the decision to vaccinate becomes more prosocial while reducing $\bar{\kappa}$ the decision becomes more self-focused. In the event a prosocial individual is infected, the individual would incur a personal cost of infection (r_I^A), but also would experience the burden of potentially infecting others in the population ($r_I \bar{\kappa}(1-\varepsilon\nu) R_0$). We captured this burden by using the effective reproductive number ($(1-\varepsilon\nu)R_0$), making use of the fact that it can be used as a proxy of the average number of secondary infections per infected individual within a population still capable of transmitting polio. Because this component involves the health of others in the population and infection of others, we valued it using the risk of paralysis due to infection among all individuals (r_I) and the parameter $\bar{\kappa}$.

The payoff to vaccinate for a prosocial is

$$-\,r_V^A - r_I \bar{\kappa} \frac{\partial \Pi(\nu, \varepsilon)}{\partial \nu}(1 - \varepsilon\nu) - (1-\varepsilon)\Pi(\nu, \varepsilon) r_I^A, \tag{S14}$$

where $\frac{\partial \Pi(\nu, \varepsilon)}{\partial \nu}$ is the marginal benefit of vaccination. The individual incurs a given risk when vaccinating due to vaccine related side effects, which is denoted $-r_V^A$. However, this risk can be offset by the effects of herd immunity ($r_V^A + r_I \bar{\kappa} \frac{\partial \Pi(\nu, \varepsilon)}{\partial \nu}(1-\varepsilon\nu)$), with the marginal benefit of vaccination approaching zero as more

people vaccinate. We add the marginal benefit of vaccination to the perceived risk to decrease the risk because $\frac{\partial \Pi(\nu,\varepsilon)}{\partial \nu} \leq 0$. Since the prosocial individual is concerned about averting transmission, we weighted the marginal benefit by the fraction of the population still capable of transmitting polio $(1-\varepsilon\nu)$. Because the marginal benefit of additional vaccination represents the change in the probability of infection with an increase in vaccination coverage, we valued the benefit using the risk of paralysis associated with infection among all individuals and the relative weight of prosocial motive $(\bar{\kappa})$. The marginal benefit is not included in equation (S13) because it is associated with a small increase in vaccination coverage and equation (S13) represents the action of not vaccinating. In the event that the vaccine is ineffective, the individual experiences only their own cost associated with infection and does not experience the burden of infecting others as they took action to prevent transmission. Furthermore, the individual would be unaware if the vaccine was effective or not. Therefore, we did not include the effective reproductive number in equation (S14). The vaccine efficacy is not included in the product $r_I\bar{\kappa}\frac{\partial \Pi(\nu,\varepsilon)}{\partial \nu}(1-\varepsilon\nu)$, (i.e. $\varepsilon r_I\bar{\kappa}\frac{\partial \Pi(\nu,\varepsilon)}{\partial \nu}(1-\varepsilon\nu)$) because $\frac{\partial \Pi(\nu,\varepsilon)}{\partial \nu}$ represents the marginal benefit of an additional vaccine, whether the vaccine is effective or not. Analytically, $\frac{\partial \Pi(\nu,\varepsilon)}{\partial \nu} = \varepsilon\frac{\partial \Pi(\nu,\varepsilon)}{\partial \phi}$, where $\phi = \varepsilon\nu$ denotes the population level of protection. Thus, including the vaccine efficacy in the product for the marginal benefit would result in accounting for the vaccine efficacy twice.

Therefore, a prosocial's expected payoff is

$$\begin{aligned} E_P(q_P,\nu,\varepsilon) &= q_P\left[-r_V^A - r_I\bar{\kappa}\frac{\partial \Pi(\nu,\varepsilon)}{\partial \nu}(1-\varepsilon\nu) - (1-\varepsilon)\Pi(\nu,\varepsilon)r_I^A\right] \\ &+ (1-q_P)\left[-\Pi(\nu,\varepsilon)\left[r_I^A + r_I\bar{\kappa}(1-\varepsilon\nu)R_0\right]\right] \end{aligned} \tag{S15}$$

Factoring out r_I^A

$$\begin{aligned} E_P(q_P,\nu,\varepsilon) &\equiv -q_P r_A - q_P\kappa\frac{\partial \Pi(\nu,\varepsilon)}{\partial \nu}(1-\varepsilon\nu) \\ &- (1-q_P\varepsilon)\Pi(\nu,\varepsilon) - (1-q_P)\Pi(\nu,\varepsilon)\kappa(1-\varepsilon\nu)R_0, \end{aligned} \tag{S16}$$

where $\kappa = \bar{\kappa}(r_I/r_I^A)$ is the strength of prosocial behavior. We assumed that $\kappa \in [0,1]$, indicating that individuals value their own health more than others [7]. Furthermore, when $r_I < r_I^A$, it indicates that an individual perceives that they are at a higher risk of paralysis than someone else in the community. Thus, one would perceive the strength of prosocial behavior to be lower because the individual is potentially at greater risk of experiencing paralysis from infection.

We denoted the risk of paralysis due to infection for an unaware individual by r_I^U and the risk of paralysis due to side effects from the vaccine by r_V^U. The steps for determining the payoffs for an unaware individual is similar to that of the individualist. Therefore, the expected payoff function for an unaware individual is

$$E_U(q_U,\nu,\varepsilon) \equiv -q_U r_U - (1-\varepsilon q_U)\Pi(\nu,\varepsilon), \tag{S17}$$

where q_U is the probability to vaccinate with OPV for an unaware individual and $r_U = r_V^U/r_I^U$ is the perceived relative risk of paralysis for an unaware individual.

Risk and perceived risk

Previous studies in Pakistan, and Nigeria found that most parents perceived paralysis as the main consequence of polio [8,9]. Thus, we only considered paralysis for the risks associated with vaccination and infection. To estimate the risk associated with paralysis, we used disability weights and the probabilities that paralysis occurs. Specifically, the risk of paralysis is the product of the disability weight and the probability of paralysis.

Disability weights are a measure of the severity of a disease on a scale from zero to one, where zero is perfect health and one is death. We estimated the value of perceived severity using disability weights for VAPP and polio paralysis. We assumed the disability weight of VAPP is comparable to severe motor impairment because there is no disability weight for VAPP. The average disability weight for severe motor impairment is 0.402 (95% CI 0.268–0.545) [10], while the disability weight for polio paralysis is 0.369 [11]. We assumed that the disability weight for VAPP is greater than or equal to the disability weight for polio paralysis when sampling the disability weight for VAPP because of omission bias—adverse outcomes are perceived to be more extreme when action is taken compared to when nothing was done [12–14]. Thus, we assumed that the disability weight for VAPP comes from a truncated Beta distribution.

For the distribution of the relative risk, we relied on the distribution of the disability weight for severe paralysis, which had a mean of 0.402 (95% CI 0.268—0.545). We assumed the disability weight follows a beta distribution ($B(a, b)$)

$$f(x|a, b) = \frac{1}{B(a, b)} x^{a-1}(1 - x)^{b-1} \tag{S18}$$

because the disability weight needs to be between zero and one. We estimated the parameters for the beta distribution by fitting the lower and upper bounds of the 95% confidence intervals. Therefore, our estimated beta distribution is B(18.7916,27.8906). We truncated the distribution such that the sampled values from the distribution were greater than the disability weight for VAPP, which is 0.369.

The probability of VAPP occurring has been estimated to occur as high as one case per 250,000 first doses of OPV [15] and as low as approximately one case per three million first doses [16]. The probability of paralysis due to polio infection is approximately one in 200 infections [17] and as low as one in a thousand infections [18]. We did not consider the reduction of the frequency of VAPP or paralysis as a result of IPV, as we assumed that vaccination with the IPV reduces the frequency of paralysis equally in these two cases of paralysis.

Using the survey data, we estimated that the perceived relative risk for an unaware individual is $r_U = \varepsilon\Pi(\varepsilon, \nu)$, where ν is the vaccination coverage, ε is the vaccine efficacy. We estimated r_U using $\varepsilon\Pi(\varepsilon, \nu)$ because the system is at NE. The results of the survey show that aware non-vaccinators were more likely to perceive OPV as unsafe compared to an unaware non-vaccinator. In addition, the survey indicates that unaware parents were more likely to vaccinate to protect their child compared to aware parents. Also, unaware vaccinators were more likely to base their decision on protecting their child than aware vaccinators. To reflect this, we assumed that $r_A > r_U$ for our analysis,implying individualist are less willing to vaccinate than unaware individuals because of the increased relative risk. If $r_A \leq r_U$ then the vaccination coverage among the aware would be greater than the vaccination coverage among the unaware. To estimate the perceived relative severity of paralysis, we maximized $(r_A - r_U)f(r_A)f(r_U)$, where $f(r)$ is the probability density function for the perceived relative risk of paralysis. We included the distance between r_A and r_U to prevent $r_A = r_U$. To construct $f(r)$, we randomly sampled the probability of VAPP occurring from its uniform distribution, the probability of paralysis due to polio from its uniform distribution, and the disability weight of VAPP from its Beta distribution to obtain the relative risk r_V/r_I, where $r_j = d_j p_j$, d_j is the disability weight, and p_j is the probability of the event occurring. We independently sampled from these distributions 1,000,000 times to construct a histogram for the relative risk of paralysis and then normalized this histogram to obtain our probability density function $f(r)$.

Distribution for R_0

A previous mathematical model estimated that the average basic reproductive number for the polio outbreak in Israel is 1.77 (95% CI 1.46—2.30) [19]. We assumed that the basic reproductive number is gamma

distributed

$$R_0 - 1 \sim \Gamma(11.923, 0.0646), \tag{S19}$$

where the probability density function for the gamma distribution ($\Gamma(a,b)$) is

$$f(x|a,b) = \frac{1}{b^A \Gamma(a)} x^{a-1} \exp\left\{\frac{x}{b}\right\} \tag{S20}$$

We estimated the parameters a and b for the gamma distribution by fitting the distribution to the average (0.77) as well as lower and upper bound of the 95% confidence interval[0.46,1.3]. We sampled from this distribution and added one to obtain the basic reproductive number for a simulation.

Quantifying the strength of prosocial behavior

We estimated a lower bound for the strength of prosocial behavior (κ) by assuming that the system is in Nash equilibrium (NE). A prosocial's difference in payoffs is

$$\left[-r_A - \kappa \frac{\partial \Pi(\nu,\varepsilon)}{\partial \nu}(1-\varepsilon\nu) - (1-\varepsilon)\Pi(\nu,\varepsilon)\right] - \left[-\Pi(\nu,\varepsilon)\left(1 + \kappa(1-\varepsilon\nu)R_0\right)\right].$$

A negative difference makes non-vaccination more appealing and we know that a prosocial prefers a higher vaccination coverage, implying this difference should be greater than or equal to zero. Thus, we estimate the minimum value for κ by setting this difference between payoffs to be zero and solving for κ. Therefore, the minimum strength of prosocial behavior is given by

$$\kappa = \frac{r_A - \varepsilon\Pi(\nu,\varepsilon)}{(1-\varepsilon\nu)\left[-\frac{\partial\Pi(\nu,\varepsilon)}{\partial\nu} + R_0\Pi(\nu,\varepsilon)\right]}, \tag{S21}$$

where ν is the observed vaccination coverage. The minimum strength of prosocial behavior will always be positive since $r_A > r_U = \varepsilon\Pi(\nu,\varepsilon) \geq \varepsilon\Pi(\overline{\nu},\varepsilon)$ for $\overline{\nu} \geq \nu$. From our analysis of the OPV campaign survey data, our model suggests that prosocial individuals always vaccinated, individualist never vaccinated, and unaware individuals vaccinated with probability q_U such that $r_U = \varepsilon\Pi(\nu,\varepsilon)$. Given a prosocial always vaccinated, they are willing to still vaccinate for a vaccination coverage ν. Given we know the prosocial equilibrium, we can substitute it into equation S21 to obtain an estimate for the strength of prosocial behavior. However, to estimate the strength of prosocial behavior without knowing the prosocial equilibrium, we defined the strength of prosocial behavior as the ratio of the benefit of vaccination to the vulnerable population to the benefit to the prosocial individual

$$\kappa = \frac{-\frac{\partial\Pi(\nu,\varepsilon)}{\partial\nu}(1-\omega)}{E_P(q_P,\varepsilon,\nu)}, \tag{S22}$$

where q_P is the NE solution for a prosocial individual and ν is the vaccination coverage at the NE solution, where individualist and unaware individuals are in the population. In the case the prosocial individual always vaccinates ($q_P = 1$), the equation simplifies to

$$\kappa = \frac{-\frac{\partial\Pi(\nu,\varepsilon)}{\partial\nu}(1-\omega)}{-r_A - \kappa\frac{\partial\Pi(\nu,\varepsilon)}{\partial\nu}(1-\varepsilon\nu) - (1-\varepsilon)\Pi(\nu,\varepsilon)}. \tag{S23}$$

Solving for κ, the estimated strength of prosocial behavior is

$$\kappa = \frac{\left[r_A + (1-\varepsilon)\Pi(\nu,\varepsilon)\right] + \sqrt{\left[r_A + (1-\varepsilon)\Pi(\nu,\varepsilon)\right]^2 + 4\left[\frac{\partial\Pi(\nu,\varepsilon)}{\partial\nu}\right]^2(1-\varepsilon\nu)(1-\omega)}}{-2\frac{\partial\Pi(\nu,\varepsilon)}{\partial\nu}(1-\varepsilon\nu)} \tag{S24}$$

Solving Nash equilibrium

To solve the Nash equilibrium (NE) for the game, we used the following algorithm.

1. For each type of individual, calculate the vaccination coverage required such that the payoff to vaccinate equals the payoff not to vaccinate. We refer to these vaccination coverages as the group equilibrium, that is individualist equilibrium, prosocial equilibrium, and unaware equilibrium
2. Sort the group equilibriums from greatest to least.
3. Pick the type of individual with the greatest group equilibrium.
4. Calculate the probability of vaccination for that type of individual (q_i) that minimizes the distance between the group equilibrium and the population vaccination coverage for groups included. For example, if only prosocails have been selected then the population vaccination uptake is $\omega\alpha\rho q_P$.
5. If the population coverage is greater than the next greatest group equilibrium then stop. Otherwise, move to the next greatest group equilibrium and repeat steps 4 and 5.

If different types of individuals require the same vaccination coverage then there is potential for multiple NE. Multiple NE can only occur when the group equilibrium can be obtained within the population and the prosocial equilibrium is the same as the unaware equilibrium. If the group equilibrium cannot be obtained then the NE is the pure strategy to vaccinate.

The algorithm implies that individuals wanting the lower vaccination coverage will free ride on others who prefer higher vaccination coverage. This free riding occurs because once vaccination coverage surpasses the group equilibrium, vaccination is no longer appealing.

Proofs for existence and uniqueness for Nash equilibrium

Show that there is a unique vaccination coverage for individualists, prosocials, and unaware individuals.

Individualist and unaware

Let q denote the probability to vaccinate for an individualist
Let p denote the fraction of the population that consists of individualist
Let ν_C denote the current vaccination coverage
Therefore, the vaccination coverage is $\nu = \nu_C + \omega pq$

Sigmoid function

For the case where $\Pi(\nu, \varepsilon) = \frac{R_0(1-\varepsilon\nu)^{1/\gamma}}{\left[R_0+(1-\varepsilon\nu)^{1/\gamma}\right]}$.
Let $f(q, p, \varepsilon, \nu) = -r_A + \varepsilon\frac{R_0(1-\varepsilon\nu)^{1/\gamma}}{\left[R_0+(1-\varepsilon\nu)^{1/\gamma}\right]}$

$$\Rightarrow \frac{\partial f}{\partial q} = -\varepsilon\frac{\partial \nu}{\partial q}\left[\varepsilon(\frac{1}{\gamma})\frac{R_0^2(1-\varepsilon\nu)^{1/\gamma-1}}{(R_0+(1-\varepsilon\nu)^{1/\gamma})^2}\right]$$

We have that $\partial\nu/\partial q \geq 0$ and $\left[\varepsilon(\frac{1}{\gamma})\frac{R_0^2(1-\varepsilon\nu)^{1/\gamma-1}}{(R_0+(1-\varepsilon\nu)^{1/\gamma})^2}\right] > 0$. Therefore, $\frac{\partial f}{\partial q} \leq 0$ for $q \in [0,1] \Rightarrow f(q,p,\varepsilon,\nu)$ is a decreasing function for $q \in [0,1]$.

Maximum prevalence

For the case where $\Pi(\nu,\varepsilon) = \frac{\ln(\frac{1}{R_0(1+\gamma)})-1+R_0(1+\gamma)(1-\varepsilon\nu)-\ln(1-\varepsilon\nu)}{R_0(1+\gamma)}$.
Let $f(q,p,\varepsilon,\nu) = -r_A + \varepsilon\frac{\ln(\frac{1}{R_0(1+\gamma)})-1+R_0(1+\gamma)(1-\varepsilon\nu)-\ln(1-\varepsilon\nu)}{R_0(1+\gamma)}$

$$\Rightarrow \frac{\partial f}{\partial q} = -\varepsilon^2\frac{\partial \nu}{\partial q}\left[-1+\frac{1}{R_0(1+\gamma)(1-\varepsilon\nu)}\right] \text{for } \nu\varepsilon \leq 1-\frac{1}{R_0(1+\gamma)} \text{ and}$$

$$\frac{\partial f}{\partial q} = 0 \text{for } \nu\varepsilon > 1-\frac{1}{R_0(1+\gamma)}$$

since $I_{max} = 0$ for $\nu\varepsilon > 1-\frac{1}{R_0(1+\gamma)}$. We have that $\partial\nu/\partial q \geq 0$ and $\left[-1+\frac{1}{R_0(1+\gamma)(1-\varepsilon\nu)}\right] < 0$ for $\nu\varepsilon \leq 1-\frac{1}{R_0(1+\gamma)}$. Therefore, $\frac{\partial f}{\partial q} \leq 0$ for $q \in [0,1] \Rightarrow f(q,p,\varepsilon,\nu)$ is a decreasing function for $q \in [0,1]$.

Polynomial

For the case where $\Pi(\nu,\varepsilon) = I_{max}(0,0,R_0)\,(1-\varepsilon\nu)^{1/\gamma}$.
Let $f(q,p,\varepsilon,\nu) = -r_A + \varepsilon\left[I_{max}(0,0,R_0)\,(1-\varepsilon\nu)^{1/\gamma}\right]$

$$\Rightarrow \frac{\partial f}{\partial q} = -\varepsilon^2 R_0\gamma\frac{\partial \nu}{\partial q}\left(\left[I_{max}(0,0,R_0)/\gamma\right](1-\varepsilon\nu)^{1/\gamma-1}\right)$$

We have that $\partial\nu/\partial q \geq 0$ and $\left[I_{max}(0,0,R_0)/\gamma\right](1-\varepsilon\nu)^{1/\gamma-1} > 0$. Therefore, $\frac{\partial f}{\partial q} \leq 0$ for $q \in [0,1] \Rightarrow f(q,p,\varepsilon,\nu)$ is a decreasing function for $q \in [0,1]$.

Estimated probability of infection

For the case where $\Pi(\nu,\varepsilon) = 1-\frac{S_\infty}{1-\varepsilon\nu}$, where S_∞ satisfies

$$0 = S_\infty + \frac{\ln(S_\infty)}{R_0} + (1-\varepsilon\nu) - \frac{\ln(1-\varepsilon\nu)}{R_0}$$

. This equation is formulated from the susceptible-infected-removed (SIR) epidemiological model

$$\begin{aligned} \frac{dS}{dt} &= -\beta SI \\ \frac{dI}{dt} &= \beta SI - \delta I \\ \frac{dR}{dt} &= \delta I \end{aligned} \tag{S25}$$

where β is the transmission rate, $1/\delta$ is the average duration of the infectious period, where $R_0 = \beta/\delta$ is the basic reproductive number where $S+I+R=1$. The partial derivative of S_∞ with respect to ν is

$$0 = -\frac{\partial S_\infty}{\partial \nu} + \frac{\partial S_\infty}{\partial \nu}\frac{1}{R_0 S_\infty} - \varepsilon + \varepsilon\frac{1}{R_0(1-\varepsilon\nu)}$$

$$\Rightarrow \frac{\partial S_\infty}{\partial \nu}\left[-1+\frac{1}{R_0 S_\infty}\right] = \varepsilon\left[1-\frac{1}{R_0(1-\varepsilon\nu)}\right]$$

$$\Rightarrow \frac{\partial S_\infty}{\partial \nu}\frac{[1-R_0 S_\infty]}{R_0 S_\infty} = \varepsilon\frac{R_0(1-\varepsilon\nu)-1}{R_0(1-\varepsilon\nu)}$$

$$\Rightarrow \frac{\partial S_\infty}{\partial \nu} = \varepsilon \frac{R_0 S_\infty}{[1 - R_0 S_\infty]} \frac{R_0(1-\varepsilon\nu) - 1}{R_0(1-\varepsilon\nu)}$$
$$\Rightarrow \frac{\partial S_\infty}{\partial \nu} = -\varepsilon \frac{R_0(1-\varepsilon\nu) - 1}{R_0 S_\infty - 1} \frac{S_\infty}{(1-\varepsilon\nu)}.$$

We have that $R_0 S_\infty - 1 < 0$, otherwise $\frac{dI}{dt} = \delta I(R_0 S_\infty - 1) > 0$ which implies the outbreak is still growing. Thus, $\frac{\partial S_\infty}{\partial \nu} > 0$ for $\varepsilon\nu \leq 1 - 1/R_0$, otherwise $\frac{\partial S_\infty}{\partial \nu} = 0$. Let $f(q,p,\varepsilon,\nu) = -r_A + \varepsilon\left[1 - \frac{S_\infty}{1-\varepsilon\nu}\right]$

$$\Rightarrow \frac{\partial f}{\partial q} = -\varepsilon \frac{\partial \nu}{\partial q}\left[\frac{\partial S_\infty}{\partial \nu}\frac{1}{1-\varepsilon\nu} + \frac{S_\infty}{(1-\varepsilon\nu)^2}\right]$$

We have that $\partial\nu/\partial q \geq 0$ and $\frac{\partial S_\infty}{\partial \nu}\frac{1}{1-\varepsilon\nu} + \frac{S_\infty}{(1-\varepsilon\nu)^2} > 0$. Therefore, $\frac{\partial f}{\partial q} \leq 0$ for $q \in [0,1] \Rightarrow f(q,p,\varepsilon,\nu)$ is a decreasing function for $q \in [0,1]$.

Case: 1

If $f(0,p,\varepsilon,\nu_C) \leq 0 \Rightarrow f(q,p,\varepsilon,\nu_C) \leq 0$ for $q \in [0,1]$ since $f(q,p,\varepsilon,\nu_C)$ is a decreasing function for $q \in [0,1]$. Therefore, the optimal strategy is not to vaccinate since the payoff not to vaccinate is always greater than the payoff to vaccinate.

Case: 2

If $f(0,p,\varepsilon,\nu_C) > 0$ and $f(1,p,\varepsilon,\nu_C) \geq 0$ then since $f(q,p,\varepsilon,\nu_C)$ is a decreasing function for $q \in [0,1]$, we know $\nexists q^* \in [0,1]$ such that $f(q^*,p,\varepsilon,\nu_C) = 0$. Therefore, the optimal strategy is to vaccinate since the payoff to vaccinate is always greater than the payoff not to vaccinate.

Case: 3

If $f(0,p,\varepsilon,\nu_C) > 0$ and $f(1,p,\varepsilon,\nu_C) < 0$ then by the intermediate value theorem $\exists q^* \in (0,1)$ such that $f(q^*,p,\varepsilon,\nu_C) = 0$. Since $f(q,p,\varepsilon,\nu_C)$ is a decreasing function for $q \in [0,1]$, this $q^* \in (0,1)$ is unique.

Assume a fraction $h \in [0,1)$ of the subpopulation is playing strategy q^*, while the remaining part of the population plays the alternative strategy $\underline{q} \neq q^*$. The payoff gain for individuals playing q^* is $f(hq^* + (1-h)\underline{q}, p, \varepsilon, \nu_C)(q^* - \underline{q})$, where $hq^* + (1-h)\underline{q}$ is the average strategy. This payoff gain is a measure for individuals to deviate from strategy $\underline{q}$ to q^*. If $\underline{q} < q^*$ then the average strategy $q = hq^* + (1-h)\underline{q} < q^* \Rightarrow f(q,p,\varepsilon,\nu_C) > 0 \Rightarrow f(hq^* + (1-h)\underline{q}, p, \varepsilon, \nu_C)(q^* - \underline{q}) > 0$. If $\underline{q} > q^*$ then the average strategy $q = hq^* + (1-h)\underline{q} > q^* \Rightarrow f(q,p,\varepsilon,\nu_C) < 0 \Rightarrow f(hq^* + (1-h)\underline{q}, p, \varepsilon, \nu_C)(q^* - \underline{q}) > 0$. Thus, q^* is a unique NE.
The proof for the unaware population is similar to that of the individualists.

Prosocial

Let q denote the probability to vaccinate for a prosocial
Let p denote the fraction of the population that consists of prosocial
Let ν_C denote the current vaccination coverage
Therefore, the vaccination coverage is $\nu = \nu_C + \omega p q$

Sigmoid function

For the case where $\Pi(\nu,\varepsilon) = \frac{R_0(1-\varepsilon\nu)^{1/\gamma}}{\left[R_0 + (1-\varepsilon\nu)^{1/\gamma}\right]}$.
Let

$$f(q,p,\varepsilon,\nu_C) \equiv -r_A + \frac{\varepsilon\kappa(1/\gamma)R_0^2(1-\varepsilon\nu)^{1/\gamma}}{\left(R_0 + (1-\varepsilon\nu)^{1/\gamma}\right)^2} + \frac{R_0(1-\varepsilon\nu)^{1/\gamma}}{\left[R_0 + (1-\varepsilon\nu)^{1/\gamma}\right]}\left(\varepsilon + \kappa(1-\varepsilon\nu)R_0\right)$$

Then,

$$\begin{aligned}\frac{\partial f}{\partial q} &= -\frac{\partial \nu}{\partial q}\varepsilon^2\kappa(R_0/\gamma)^2\frac{R_0-(1-\varepsilon\nu)^{1/\gamma}}{\left[R_0+(1-\varepsilon\nu)^{1/\gamma}\right]^3}\\ &- \frac{\partial \nu}{\partial q}\left[\varepsilon+\kappa(1-\varepsilon\nu)R_0\right]\left[\frac{\varepsilon\kappa(1/\gamma)R_0^2(1-\varepsilon\nu)^{1/\gamma-1}}{\left(R_0+(1-\varepsilon\nu)^{1/\gamma}\right)^2}\right]\\ &- \frac{\partial \nu}{\partial q}\kappa\varepsilon R_0\left[\frac{R_0(1-\varepsilon\nu)^{1/\gamma}}{\left[R_0+(1-\varepsilon\nu)^{1/\gamma}\right]}\right].\end{aligned}$$

We have that $\frac{\partial \nu}{\partial q} \geq 0$, $R_0 > 1 \Rightarrow R_0-(1-\varepsilon\nu)^{1/\gamma} > 0$ and $\frac{\varepsilon\kappa(1/\gamma)R_0^2(1-\varepsilon\nu)^{1/\gamma-1}}{(R_0+(1-\varepsilon\nu)^{1\gamma})^2} > 0$. Therefore, $\frac{\partial f}{\partial q} \leq 0$ for $q \in [0,1]$, which implies that $f(q,p,\varepsilon,\nu_C)$ is a decreasing function for $q \in [0,1]$.

Maximum prevalence

For the case where $\Pi(\nu,\varepsilon) = \frac{\ln(\frac{1}{R_0(1+\gamma)})-1+R_0(1+\gamma)(1-\varepsilon\nu)-\ln(1-\varepsilon\nu)}{R_0(1+\gamma)}$.
Let

$$\begin{aligned}f(q,p,\varepsilon,\nu_C) &\equiv -r_A+\kappa\varepsilon\left[(1-\varepsilon\nu)-\frac{1}{R_0(1+\gamma)}\right]\\ &+ \frac{\ln(\frac{1}{R_0(1+\gamma)})-1+R_0(1+\gamma)(1-\varepsilon\nu)-\ln(1-\varepsilon\nu)}{R_0(1+\gamma)}\left[\varepsilon+\kappa(1-\varepsilon\nu)R_0\right]\end{aligned}$$

Then,

$$\begin{aligned}\frac{\partial f}{\partial q} &= -\frac{\partial \nu}{\partial q}\varepsilon^2\kappa\\ &- \frac{\partial \nu}{\partial q}\varepsilon^2\frac{R_0(1+\gamma)(1-\varepsilon\nu)-1}{R_0(1+\gamma)(1-\varepsilon\nu)}\\ &- \frac{\partial \nu}{\partial q}\varepsilon^2\kappa\frac{\ln(\frac{1}{R_0(1+\gamma)})-1+R_0(1+\gamma)(1-\varepsilon\nu)-\ln(1-\varepsilon\nu)}{R_0(1+\gamma)}\end{aligned}$$

We have $\frac{\partial \nu}{\partial q} > 0$. For for $\varepsilon\nu > 1-1/(R_0(1+\gamma))$: we have $\Pi(\nu,\varepsilon) = 0 \Rightarrow \partial\Pi(\nu,\varepsilon)/\partial\nu = 0$. For $\varepsilon\nu \leq 1-1/(R_0(1+\gamma)) \Rightarrow (1-\varepsilon\nu)R_0(1+\gamma)$ then $\frac{\partial f}{\partial q} \leq 0$ for $q \in [0,1] \Rightarrow f(q,p,\varepsilon,\nu_C)$ is a decreasing function for $q \in [0,1]$.

Polynomial

For the case where $\Pi(\nu,\varepsilon) = I_{max}(0,0,R_0)\left(1-\varepsilon\nu\right)^{1/\gamma}$.
Let

$$\begin{aligned}f(q,p,\varepsilon,\nu) &= -r_A+\kappa\varepsilon\frac{I_{max}(0,0,R_0)}{\gamma}\left(1-\varepsilon\nu\right)^{1/\gamma-1}\left(1-\varepsilon\nu\right)\\ &+ \left[I_{max}(0,0,R_0)\left(1-\varepsilon\nu\right)^{1/\gamma}\right]\left[\varepsilon+\kappa R_0(1-\varepsilon\nu)\right]\end{aligned}$$

Then,

$$\begin{aligned}\frac{\partial f}{\partial q} &= -\frac{\partial \nu}{\partial q}\varepsilon^2\kappa\frac{I_{max}(0,0,R_0)}{\gamma^2}\left(1-\varepsilon\nu\right)^{1/\gamma-1}\\ &- \frac{\partial \nu}{\partial q}\varepsilon\frac{I_{max}(0,0,R_0)}{\gamma}\left(1-\varepsilon\nu\right)^{1/\gamma-1}\left[\varepsilon+\kappa R_0(1-\varepsilon\nu)\right]\\ &- \frac{\partial \nu}{\partial q}R_0\kappa\varepsilon\left[I_{max}(0,0,R_0)\left(1-\varepsilon\nu\right)^{1/\gamma}\right]\end{aligned}$$

Since $(1-\varepsilon\nu)^{1/\gamma-1} \geq 0$, $(1-\varepsilon\nu)^{1/\gamma} \geq 0$, and $\frac{\partial \nu}{\partial q} \geq 0$ then we have that $\frac{\partial f}{\partial q} \leq 0$. Thus, since $\frac{\partial \nu}{\partial q} \geq 0$ for $q \in [0,1] \Rightarrow f(q,p,\varepsilon,\nu)$ is a decreasing function for $q \in [0,1]$.

True probability of infection

For the case where $\Pi(\nu,\varepsilon) = 1 - \frac{S_\infty}{1-\varepsilon\nu}$. We have that

$$\frac{\partial S_\infty}{\partial \nu} = -\varepsilon \frac{S_\infty}{(1-\varepsilon\nu)} \frac{R_0(1-\varepsilon\nu)-1}{R_0 S_\infty - 1}.$$

Thus,

$$\begin{aligned}
\frac{\partial^2 S_\infty}{\partial \nu^2} &= -\varepsilon \frac{\partial S_\infty}{\partial \nu} \frac{1}{1-\varepsilon\nu)} \frac{R_0(1-\varepsilon\nu)-1}{R_0 S_\infty - 1} \\
&- \varepsilon^2 \frac{S_\infty}{(1-\varepsilon\nu)^2} \frac{R_0(1-\varepsilon\nu)-1}{R_0 S_\infty - 1} \\
&+ \varepsilon^2 \frac{S_\infty}{(1-\varepsilon\nu)} \frac{R_0}{R_0 S_\infty - 1} \\
&+ \frac{\partial S_\infty}{\partial \nu} \varepsilon \frac{S_\infty}{(1-\varepsilon\nu)} \frac{R_0^2(1-\varepsilon\nu)-1}{(R_0 S_\infty - 1)^2} \\
\Rightarrow \frac{\partial^2 S_\infty}{\partial \nu^2} &= \left(\frac{\partial S_\infty}{\partial \nu}\right)^2 \frac{1}{S_\infty} + \left(\frac{\partial S_\infty}{\partial \nu}\right) \frac{\varepsilon}{1-\varepsilon\nu} \\
&- \left(\frac{\partial S_\infty}{\partial \nu}\right) \frac{\varepsilon R_0}{R_0(1-\varepsilon\nu)-1} + \left(\frac{\partial S_\infty}{\partial \nu}\right)^2 \frac{R_0}{R_0 S_\infty - 1} \\
\Rightarrow \frac{\partial^2 S_\infty}{\partial \nu^2} &= \left(\frac{\partial S_\infty}{\partial \nu}\right)^2 \left(\frac{1}{S_\infty} - \frac{R_0}{R_0 S_\infty - 1}\right) \\
&+ \varepsilon \left(\frac{\partial S_\infty}{\partial \nu}\right) \left(\frac{1}{1-\varepsilon\nu} - \frac{R_0}{R_0(1-\varepsilon\nu)-1}\right) \\
\Rightarrow \frac{\partial^2 S_\infty}{\partial \nu^2} &= -\left(\frac{\partial S_\infty}{\partial \nu}\right)^2 \frac{1}{S_\infty(R_0 S_\infty - 1)} - \varepsilon \left(\frac{\partial S_\infty}{\partial \nu}\right) \frac{1}{(1-\varepsilon\nu)(R_0(1-\varepsilon\nu)-1)} \\
\Rightarrow \frac{\partial^2 S_\infty}{\partial \nu^2} &= \left(\frac{\partial S_\infty}{\partial \nu}\right) \left(\frac{\varepsilon((R_0(1-\varepsilon\nu)-1))}{(1-\varepsilon\nu)(R_0 S_\infty - 1)^2} - \frac{\varepsilon}{(1-\varepsilon\nu)(R_0(1-\varepsilon\nu)-1)}\right) \\
\Rightarrow \frac{\partial^2 S_\infty}{\partial \nu^2} &= \varepsilon \left(\frac{\partial S_\infty}{\partial \nu}\right) \left(\frac{(R_0(1-\varepsilon\nu)-1)^2 - (R_0 S_\infty - 1)^2}{(1-\varepsilon\nu)(R_0(1-\varepsilon\nu)-1)(R_0 S_\infty - 1)^2}\right)
\end{aligned}$$

We know that $R_0 S_\infty - 1 < 0$ and $R_0(1-\varepsilon\nu) - 1 > 0$. Assume that $1 - R_0 S_\infty > R_0(1-\varepsilon\nu) - 1 \; \forall S_\infty < (1-\varphi)$. We have that S_∞ satisfies

$$0 = -S_\infty + \ln(S_\infty)/R_0 + (1-\varepsilon\nu) - \ln(1-\varepsilon\nu)/R_0$$

$$\Rightarrow 0 = -R_0 S_\infty + \ln(S_\infty) - \ln(1-\varepsilon\nu) + R_0(1-\varepsilon\nu)$$

$$\Rightarrow 0 = 1 - R_0 S_\infty + \ln(S_\infty/(1-\varepsilon\nu)) + R_0(1-\varepsilon\nu) - 1$$

Since we assumed that $1 - R_0 S_\infty > R_0(1-\varepsilon\nu) - 1$ then

$$1 - R_0 S_\infty + \ln(S_\infty/(1-\varepsilon\nu)) + R_0(1-\varepsilon\nu) - 1 \leq 2(1 - R_0 S_\infty) + \ln(S_\infty/(1-\varepsilon\nu)) \forall S_\infty < (1-\varphi)$$

We have that $S_\infty = (1-\varepsilon\nu)$ satisfies $0 = -R_0 S_\infty + \ln(S_\infty) - \ln(1-\varepsilon\nu) + R_0(1-\varepsilon\nu)$ and $-R_0 S_\infty + \ln(S_\infty) - \ln(1-\varepsilon\nu) + R_0(1-\varepsilon\nu)$ is an increasing function with respect to S_∞. As $S_\infty \to 0^+$ then $R_0 \to \infty$

which implies that $-S_\infty R_0 = C$ where $C \in (-1, 0)$ since $R_0 S_\infty - 1 < 0 \Rightarrow -1 < -R_0 S_\infty < 0$. In addition, as $S_\infty \to 0^+$ then $\ln(S_\infty/(1-\varepsilon\nu)) \to -\infty$. Thus,

$$2(1 - R_0 S_\infty) + \ln(S_\infty/(1-\varepsilon\nu)) \to -\infty \forall S_\infty < (1-\varphi)$$

for $S_\infty \to 0^+$ which contradicts $2(1 - R_0 S_\infty) + \ln(S_\infty/(1-\varepsilon\nu)) \forall S_\infty < (1-\varphi) \geq 0$ for $S_\infty < 1 - \varepsilon\nu$. Thus, $0 < 1 - R_0 S_\infty < R_0(1-\varepsilon\nu) - 1$ which implies that $(1 - R_0 S_\infty)^2 < (R_0(1-\varepsilon\nu)-1)^2$. Therefore, $\frac{\partial^2 S_\infty}{\partial \nu^2} \geq 0$. Let

$$f(q, p, \varepsilon, \nu) = -r_A + \kappa \left(\frac{\partial S_\infty}{\partial \nu} + \varepsilon \frac{S_\infty}{1 - \varepsilon\nu} \right) + [1 - S_\infty/(1-\varepsilon\nu)] \left[\varepsilon + \kappa(1-\varepsilon\nu)R_0 \right]$$

then

$$\begin{aligned}
\frac{\partial f}{\partial q} &= (\frac{\partial \nu}{\partial q})\kappa \left[\frac{\partial^2 S_\infty}{\partial \nu^2} + \varepsilon \frac{\partial S_\infty}{\partial \nu}/(1-\varepsilon\nu) + \varepsilon^2 S_\infty/(1-\varepsilon\nu)^2 \right] \\
&- (\frac{\partial \nu}{\partial q}) \left[\frac{\partial S_\infty}{\partial \nu}/(1-\varepsilon\nu) + \varepsilon S_\infty/(1-\varepsilon\nu)^2 \right] \left[\varepsilon + \kappa(1-\varepsilon\nu)R_0 \right] \\
&- (\frac{\partial \nu}{\partial q})[1 - S_\infty/(1-\varepsilon\nu)]\kappa\varepsilon R_0 \\
&= (\frac{\partial \nu}{\partial q})\kappa \left[\frac{\partial^2 S_\infty}{\partial \nu^2} + \frac{\partial S_\infty}{\partial \nu}/(1-\varepsilon\nu) \left(\varepsilon - (1 - \varepsilon R_0)\right) + \varepsilon S_\infty/(1-\varepsilon\nu)^2 \left(\varepsilon - (1-\varepsilon\nu)R_0\right) \right] \\
&- \varepsilon(\frac{\partial \nu}{\partial q}) \left[\frac{\partial S_\infty}{\partial \nu}/(1-\varepsilon\nu) + \varepsilon S_\infty/(1-\varepsilon\nu)^2 \right] \\
&- (\frac{\partial \nu}{\partial q})[1 - S_\infty/(1-\varepsilon\nu)]\kappa\varepsilon R_0 \\
&\leq (\frac{\partial \nu}{\partial q})\kappa \left[\frac{\partial^2 S_\infty}{\partial \nu^2} + \frac{\partial S_\infty}{\partial \nu}/(1-\varepsilon\nu) \left(1 - (1 - \varepsilon R_0)\right) + \varepsilon S_\infty/(1-\varepsilon\nu)^2 \left(1 - (1-\varepsilon\nu)R_0\right) \right] \\
&- \varepsilon(\frac{\partial \nu}{\partial q}) \left[\frac{\partial S_\infty}{\partial \nu}/(1-\varepsilon\nu) + \varepsilon S_\infty/(1-\varepsilon\nu)^2 \right] \\
&- (\frac{\partial \nu}{\partial q})[1 - S_\infty/(1-\varepsilon\nu)]\kappa\varepsilon R_0 \\
&= (\frac{\partial \nu}{\partial q})\kappa \left[\frac{\partial^2 S_\infty}{\partial \nu^2} - \frac{\partial S_\infty}{\partial \nu}/(1-\varepsilon\nu) \left(R_0(1 - \varepsilon - 1)\right) \right] \\
&- (\frac{\partial \nu}{\partial q}) \left[\varepsilon \frac{S_\infty}{(1-\varepsilon\nu)} \frac{(R_0(1-\varepsilon\nu)R - 1)}{1 - R_0 S_\infty} \frac{1 - R_0 S_\infty}{(1-\varepsilon\nu)} \right] \\
&- \varepsilon(\frac{\partial \nu}{\partial q}) \left[\frac{\partial S_\infty}{\partial \nu}/(1-\varepsilon\nu) + \varepsilon S_\infty/(1-\varepsilon\nu)^2 \right] \\
&- (\frac{\partial \nu}{\partial q})[1 - S_\infty/(1-\varepsilon\nu)]\kappa\varepsilon R_0 \\
&= (\frac{\partial \nu}{\partial q})\kappa \left[\frac{\partial^2 S_\infty}{\partial \nu^2} - \frac{\partial S_\infty}{\partial \nu}/(1-\varepsilon\nu) \left(R_0(1 - \varepsilon - 1)\right) - \frac{\partial S_\infty}{\partial \nu} \frac{1 - R_0 S_\infty}{(1-\varepsilon\nu)} \right] \\
&- \varepsilon(\frac{\partial \nu}{\partial q}) \left[\frac{\partial S_\infty}{\partial \nu}/(1-\varepsilon\nu) + \varepsilon S_\infty/(1-\varepsilon\nu)^2 \right] \\
&- (\frac{\partial \nu}{\partial q})[1 - S_\infty/(1-\varepsilon\nu)]\kappa\varepsilon R_0
\end{aligned}$$

Since we have that

$$\begin{aligned}
\frac{\partial^2 S_\infty}{\partial \nu^2} &= \varepsilon \left(\frac{\partial S_\infty}{\partial \nu}\right)\left(\frac{(R_0(1-\varepsilon\nu)-1)^2-(R_0S_\infty-1)^2}{(1-\varepsilon\nu)(R_0(1-\varepsilon\nu)-1)(R_0S_\infty-1)^2}\right)\\
&\leq \left(\frac{\partial S_\infty}{\partial \nu}\right)\left(\frac{(R_0(1-\varepsilon\nu)-1)^2-(R_0S_\infty-1)^2}{(1-\varepsilon\nu)(R_0(1-\varepsilon\nu)-1)(R_0S_\infty-1)^2}\right)
\end{aligned}$$

then

$$\begin{aligned}
\frac{\partial f}{\partial q} &\leq (\frac{\partial \nu}{\partial q})\kappa\left[\left(\frac{\partial S_\infty}{\partial \nu}\right)\left(\frac{(R_0(1-\varepsilon\nu)-1)^2-(R_0S_\infty-1)^2}{(1-\varepsilon\nu)(R_0(1-\varepsilon\nu)-1)(R_0S_\infty-1)^2}\right)\right]\\
&- (\frac{\partial \nu}{\partial q})\kappa\left[\frac{\partial S_\infty}{\partial \nu}/(1-\varepsilon\nu)\left(R_0(1-\varepsilon-1)\right)+\frac{\partial S_\infty}{\partial \nu}\frac{1-R_0S_\infty}{(1-\varepsilon\nu)}\right]\\
&- \varepsilon(\frac{\partial \nu}{\partial q})\left[\frac{\partial S_\infty}{\partial \nu}/(1-\varepsilon\nu)+\varepsilon S_\infty/(1-\varepsilon\nu)^2\right]\\
&- (\frac{\partial \nu}{\partial q})[1-S_\infty/(1-\varepsilon\nu)]\kappa\varepsilon R_0\\
&= (\frac{\partial \nu}{\partial q})\kappa\left[\left(\frac{\partial S_\infty}{\partial \nu}\right)\frac{\{(R_0(1-\varepsilon\nu)-1)+(1-R_0S_\infty)\}\{(R_0(1-\varepsilon\nu)-1)-(1-R_0S_\infty)\}}{(1-\varepsilon\nu)(R_0(1-\varepsilon\nu)-1)(R_0S_\infty-1)^2}\right]\\
&- (\frac{\partial \nu}{\partial q})\kappa\frac{\frac{\partial S_\infty}{\partial \nu}}{(1-\varepsilon\nu)}\left[(R_0(1-\varepsilon\nu)-1))+(1-R_0S_\infty)\right]\\
&- \varepsilon(\frac{\partial \nu}{\partial q})\left[\frac{\partial S_\infty}{\partial \nu}/(1-\varepsilon\nu)+\varepsilon S_\infty/(1-\varepsilon\nu)^2\right]\\
&- (\frac{\partial \nu}{\partial q})[1-S_\infty/(1-\varepsilon\nu)]\kappa\varepsilon R_0\\
&= (\frac{\partial \nu}{\partial q})\kappa\left[\left(\frac{\partial S_\infty}{\partial \nu}\right)\frac{\{(R_0(1-\varepsilon\nu)-1)+(1-R_0S_\infty)\}\{(R_0(1-\varepsilon\nu)-1)-(1-R_0S_\infty)\}}{(1-\varepsilon\nu)(R_0(1-\varepsilon\nu)-1)(R_0S_\infty-1)^2}\right]\\
&- (\frac{\partial \nu}{\partial q})\kappa\frac{\frac{\partial S_\infty}{\partial \nu}}{(1-\varepsilon\nu)}\left[(R_0(1-\varepsilon\nu)-1))+(1-R_0S_\infty)\right]\frac{(R_0(1-\varepsilon\nu)-1)(R_0S_\infty-1)^2}{(R_0(1-\varepsilon\nu)-1)(R_0S_\infty-1)^2}\\
&- \varepsilon(\frac{\partial \nu}{\partial q})\left[\frac{\partial S_\infty}{\partial \nu}/(1-\varepsilon\nu)+\varepsilon S_\infty/(1-\varepsilon\nu)^2\right]\\
&- (\frac{\partial \nu}{\partial q})[1-S_\infty/(1-\varepsilon\nu)]\kappa\varepsilon R_0
\end{aligned}$$

$$\begin{aligned}
&= (\frac{\partial \nu}{\partial q})\frac{\kappa\frac{\partial S_\infty}{\partial \nu}\left[(R_0(1-\varepsilon\nu)-1))+(1-R_0S_\infty)\right]}{(1-\varepsilon\nu)(R_0(1-\varepsilon\nu)-1)(R_0S_\infty-1)^2}\\
&\cdot \left((R_0(1-\varepsilon\nu)-1)-(1-R_0S_\infty)-(R_0(1-\varepsilon\nu)-1)(1-R_0S_\infty)^2\right)\\
&- \varepsilon(\frac{\partial \nu}{\partial q})\left[\frac{\partial S_\infty}{\partial \nu}/(1-\varepsilon\nu)+\varepsilon S_\infty/(1-\varepsilon\nu)^2\right]\\
&- (\frac{\partial \nu}{\partial q})[1-S_\infty/(1-\varepsilon\nu)]\kappa\varepsilon R_0\\
&= (\frac{\partial \nu}{\partial q})\frac{\kappa\frac{\partial S_\infty}{\partial \nu}\left[(R_0(1-\varepsilon\nu)-1))+(1-R_0S_\infty)\right]}{(1-\varepsilon\nu)(R_0(1-\varepsilon\nu)-1)(R_0S_\infty-1)^2}\left((R_0(1-\varepsilon\nu)-1)(1-(1-R_0S_\infty)^2)-(1-R_0S_\infty)\right)\\
&- \varepsilon(\frac{\partial \nu}{\partial q})\left[\frac{\partial S_\infty}{\partial \nu}/(1-\varepsilon\nu)+\varepsilon S_\infty/(1-\varepsilon\nu)^2\right]\\
&- (\frac{\partial \nu}{\partial q})[1-S_\infty/(1-\varepsilon\nu)]\kappa\varepsilon R_0
\end{aligned}$$

Show that $(R_0(1-\varepsilon\nu)-1)(1-(1-R_0S_\infty)^2)-(1-R_0S_\infty)\leq 0$ for $R_0(1-\varepsilon\nu)\geq 1$. If $0<R_0(1-\varepsilon\nu)<1$ then $\frac{\partial S_\infty}{\partial \nu}=\frac{\partial^2 S_\infty}{\partial \nu^2}=0$, which implies that $\partial f/\partial q\leq 0$.

Let $g(S_\infty)=(R_0(1-\varepsilon\nu)-1)(1-(1-R_0S_\infty)^2)-(1-R_0S_\infty)$ then

$$\frac{\partial g}{\partial S_\infty}=(R_0(1-\varepsilon\nu)-1)(2R_0(1-R_0S_\infty))+R_0\geq 0$$

Thus, $g(S_\infty)$ is an increasing function with respect to S_∞. We know that $S_\infty\in(0,1-\varepsilon\nu]$. Thus,

$$g(0)=(R_0(1-\varepsilon\nu)-1)(1-(1)^2)-1=-1$$

For $S_\infty=1-\varepsilon\nu$ the $R_0(1-\varepsilon\nu)=1$ since $R_0(1-\varepsilon\nu)\geq 1$ and if $R_0(1-\varepsilon\nu)>1$ then infection would expand ($dI/dt>0$) in the population and $S_\infty<1-\varepsilon\nu$. Thus,

$$g(1-\varepsilon\nu)=(R_0(1-\varepsilon\nu)-1)(1-(1-R_0(1-\varepsilon\nu))^2)-(1-R_0(1-\varepsilon\nu))=0.$$

Therefore, $(R_0(1-\varepsilon\nu)-1)(1-(1-R_0S_\infty)^2)-(1-R_0S_\infty)\leq 0$ for $R_0(1-\varepsilon\nu)\geq 1$. Thus, $\frac{\partial f}{\partial q}\leq 0$ for $q\in[0,1]$ which implies that $f(q,\varepsilon,\nu)$ is a decreasing function for $q\in[0,1]$.

The three cases for the prosocial individual is similar to the three cases proved for the individualist and unaware individual.

Proof aware prosocial individual more likely to vaccinate than aware self-interested individual

We use proof by contradiction to show that $q_P\geq q_S$. Assume that the prosocial equilibrium (i.e. payoff not to vaccinate equals the payoff to vaccinate) is less than or equal to the individualist equilibrium, i.e. $\nu_P<\nu_S$. This assumption implies that $\varepsilon\omega q_P<\varepsilon\omega q_S\Rightarrow q_P<q_S$ Let $f(q)=-r_A-\kappa\frac{\partial\Pi(\varepsilon,\nu)}{\partial\nu}(1-\varepsilon\nu)+\Pi(\varepsilon,\nu)\left[\varepsilon+\kappa(1-\varepsilon\nu)R_0\right]$ for the difference in payoff for a prosocial and $g(q)=-r_A+\varepsilon\Pi(\varepsilon,\nu)$ be the difference in payoff for an individualist. We have that $f(q_P)=0$ and that $g(q_S)=0$. We have shown previously in the NE section that both f and g are decreasing functions. Since $q_P<q_S$ then $f(q_P)>f(q_S)\Rightarrow 0>f(q_S)$. Because $r_A=\varepsilon\Pi(\varepsilon,\nu)$ for $q=q_S$ then $f(q_S)=-\kappa\frac{\partial\Pi(\varepsilon,\nu)}{\partial\nu}(1-\varepsilon\nu)+\Pi(\varepsilon,\nu)\kappa(1-\varepsilon\nu)R_0$. Since $\frac{\partial\Pi(\varepsilon,\nu)}{\partial\nu}\leq 0$ and $\Pi(\varepsilon,\nu)\geq 0$ then $f(q_S)\geq 0$, which is a contradiction. Therefore, an aware prosocial individual is more likely to vaccinate than an individualist, i.e., $q_P\geq q_S$.

Likelihood calculation

To estimate the likelihood of the vaccination coverage being 72%, we sampled 2,500 basic reproductive numbers and 2,500 disability weights and determined the vaccination coverage from the 2,500 samples. We then binned each vaccination coverage into 1% intervals from 0% to 100% using MATLAB's hist function and normalized the output. We then determined the likelihood that the vaccination coverage was 72%.

Validation

For validation of our model, we estimated the degree of prosociality and the sensitivity to infection for five sociocultural groups in Israel. We estimated the fraction of the population that consisted of prosocial individuals among the five different sociocultural groups (Table 1, Table S9-S11). To determine the density of prosocials among the aware population (ρ), we assumed that an unaware individual is 1.224 times more likely to vaccinate compared to aware individuals, consistent with our baseline model calibration for Israel. Without this assumption and not using the vaccination coverage among the aware/unaware (as we are

validating the findings regarding behavior), one could obtain a large range of estimates for the density of prosocials among the aware population (ρ). We based these assumptions on findings from the survey study that indicated that people who agreed with the statement that the vaccine was given to stop transmission and not necessarily to protect the individual (i.e. comprehended the prosocial nature of the campaign) were 1.224 times more likely to vaccinate than those who disagreed. The vaccination coverage is expressed as $\nu = \omega(\alpha\rho q_P + \alpha(1-\rho)q_S + (1-\alpha)q_U) = (\alpha\nu_A + (1-\alpha)\nu_U)$, where $\nu_A = \omega(\alpha\rho q_P + \alpha(1-\rho)q_S)$ is the vaccination coverage among the aware and $\nu_U = \omega q_U$ is the vaccination coverage among the unaware. We found that for the OPV campaign in Israel for the baseline parameters that individualist never vaccinated ($q_S = 0$) and prosocials always vaccinated ($q_P = 1$). Thus, the vaccination coverage for the aware is $\nu_A = \omega\rho$. Since we assumed that $\nu_U = 1.224\nu_A$ then $\nu_U = 1.224\omega\rho$. Thus, the vaccination coverage is approximated by $\nu = \omega\rho\alpha + 1.224\omega\rho(1-\alpha) = \omega\rho(\alpha + 1.224(1-\alpha)) = \omega\rho(1.224 - 0.224\alpha))$. Given the overall vaccination coverage, the proportion of the population that comprehended the nature of the campaign (α), and the proportion eligible for vaccination (ω), the approximated prosocial density of prosocials is

$$\rho = \frac{\nu}{\omega\,(1.224 - 0.224\alpha)}.$$

Alternatively, given the vaccination coverage among the aware (ν_A) is known then the estimate for the proportion of aware people that are prosocial is $\rho = \frac{\nu_A}{\omega}$.Our approximation of the degree of prosociality produced results consistent with estimates from a previous survey study among the five socio-cultural groups (Table S12).

Sensitivity analysis

We conducted sensitivity analysis on the perceived basic reproductive number (R_0), the perceived efficacy of the OPV (ε), the fraction of the population eligible for vaccination (ω), the relative magnitude of risk between aware and unaware individuals (r_A vs r_U). We randomly and independently sampled each parameter from their specified distribution to obtain 5,000 parameter sets. For each parameter set, we estimated the sensitivity to infection, the degree of prosociality, and the strength of prosociality. We then fixed a parameter (e.g. R_0) to its baseline value and re-ran the model with the 5,000 other parameter combinations to determine the influence on the distribution of the estimated outcomes. To examine the changes in the distribution for a given fixed parameter, we examined the changes not only in the mean and standard deviation but estimated the difference in the overall distribution by calculating the area between the curves where the baseline distribution is greater than the fixed parameter distribution:

$$\Delta = \int_{-\infty}^{\infty} (g(x) - f(x))\, H(g(x) - f(x))dx, \tag{S26}$$

where $g(x)$ is the baseline probability density function for the estimate, $f(x)$ is the fixed parameter probability density function for the estimate, and

$$H(x) = \begin{cases} 1 & x > 0 \\ 0 & x \leq 0 \end{cases}. \tag{S27}$$

The area between the curves, $\Delta \approx 0$ indicates that fixing that parameter has little impact on the outcome, whereas greater values of Δ indicate that more information pertaining to that parameter could provide a more accurate estimate of the outcome. For example, knowing the difference between the relative risk of the aware and unaware will provide a more accurate estimate of the strength of prosocial behavior for the sigmodial function (Figure S6-S9). We found that the estimate of the proportion of prosocial behavior among aware individuals was most impacted by the level of comprehension in the population and the proportion of the individuals eligible to receive the OPV. These parameters directly influence the overall

level of prosociality within the population, as a lower level of comprehension or fewer people eligible would require a higher proportion of aware individuals to behave prosocially. The strength of prosocial behavior was influenced by the difference between the relative risk of the aware and unaware for the sigmoidal function and maximum prevalence. We see a similar effect among the three probabilities of infection on the minimum estimate of the strength of prosocial behavior. Assuming greater risk among the aware individuals requires a greater strength of prosocial behavior to offset this high-risk.

Code availability

The computational code used to produce these results can be found at
https://github.com/WellsRC/Israel_OPV_2013.

Table S8: Baseline parameters used in the model simulations.

**Parameter**	**Description**	**Value (Range)**	**Reference**
R_0	Basic reproductive number	1.77 (1.40–2.26)	[19][a]
R_0^P	Perceived basic reproductive number	2.24	Assumed [b]
ε	Vaccine efficacy of a single dose of OPV	0.63 (0.57–1)[c]	[19,20]
r_U	Relative risk of paralysis for unaware	2.14×10^{-4}	Estimated [d]
r_A	Relative risk of paralysis for aware	1×10^{-3}	Estimated [d]
ω	The proportion of the population vaccinated with IPV	0.94 (0.90–0.98)	[21][e]
α	The proportion of the population that comprehended campaign had pure prosocial motive	0.548	
ρ	The proportion of the aware population that is prosocial	0.697	Estimated [f]

a The range is based on the 95% confidence interval of a gamma distribution fit to the 95% confidence interval (1.46–2.30) and mean (1.77) of the basic reproductive number from [19]

b The perceived basic reproductive number was based on the estimate of the perceived basic reproductive number from the Arab population using the estimated probability of infection

c Lower bound is based on average estimate from [20]

d The relative risk of paralysis was assumed to be the value that maximized the distance between r_A and r_U as well as maximize the likelihood

e The IPV coverage is based on the 2012 coverage, as the outbreak occurred in 2013

f The percentage of the aware population that is prosocial was estimated such that the fraction of eligible prosocial individuals eligible for vaccination was 65.5% ($\rho\omega = 0.655$).

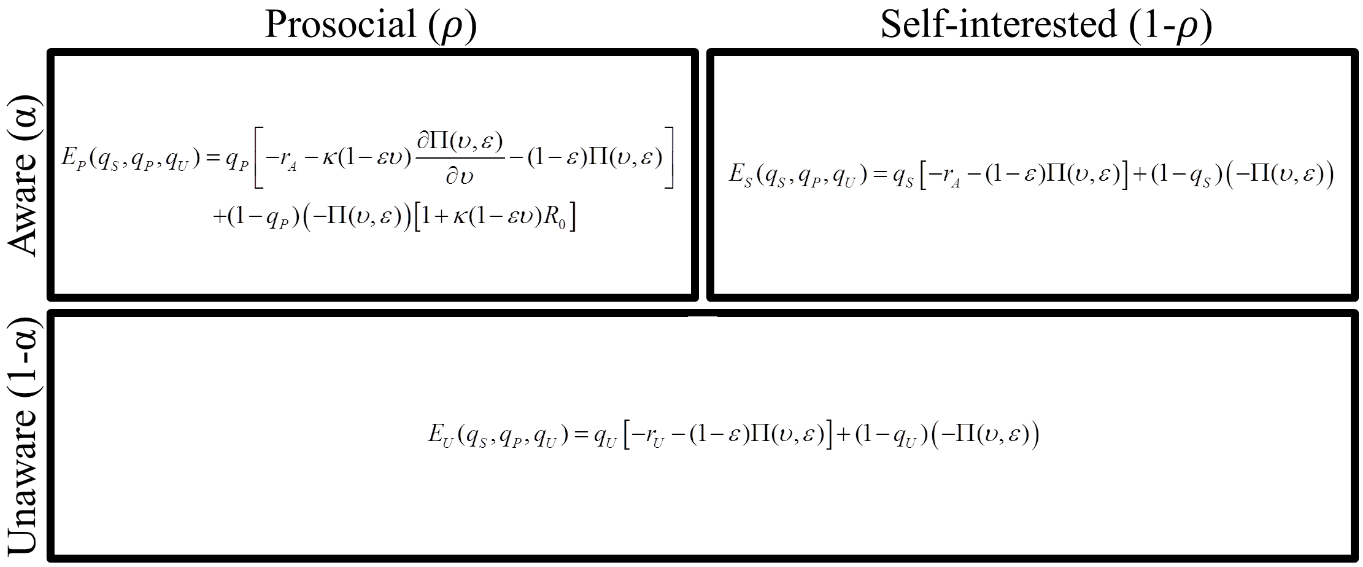

Figure S1: Schematic of the model population stratification with the corresponding expected payoff functions. The population includes the prosocial (prosocial and aware (top left)), the individualist (self-interested and aware (top right)), and the unaware (prosocial/self-interested and unaware (bottom)). Prosocials vaccinate with probability q_P, individualist with probability q_S, and unaware with probability q_U.

Table S9: The degree of prosociality during the 2013 OPV vaccination campaign estimated from the game theory model and results from the survey under the maximum prevalence probability of infection $(I_{\max}(R_0(1+\gamma), \varepsilon\nu)^a$.

	**National**	**Orthodox**	**Religious**	**Traditional**	**Secular**	**Arab**
Vaccination coverage (ν)	72.1%	78.1%	63.6%	72.3%	63.7%	88.0%
Comprehended campaign as prosocial (α)	54.8%	48.4%	56.2%	63.6%	66.8%	25.5%
Estimated aware that are prosocial (ρ) ^{b}	69.7%	74.5%	61.6%	71.1%	63.1%	80.2%
Minimum strength of prosocial behavior (κ)	0.063	0.065	0.060	0.063	0.060	0.070
Estimate strength of prosocial behavior $(\kappa)^c$	0.38	0.40	0.36	0.38	0.36	0.42
Sensitivity to infection (γ)	-0.152	-0.088	-0.229	-0.150	-0.228	0.049

a We assumed a vaccine efficacy of 63%, 94% of the population is eligible for vaccination, the perceived basic reproductive number to be 2.24, the relative risk for an unaware individuals to be approximately 2.14×10^{-4}, the relative risk for an aware individual to be 0.001.

b The percentage of the eligible population that is prosocial was estimated based on the assumption that unaware individuals were approximately 1.22 times more likely to vaccinate than an aware individual (SI)

c Estimated using equation S24

Table S10: The degree of prosociality during the 2013 OPV vaccination campaign estimated from the game theory model and results from the survey under the polynomial probability of infection $(I_{\max}(R_0, 0)(1 - \varepsilon\nu)^{1/\gamma})^a$.

	**National**	**Orthodox**	**Religious**	**Traditional**	**Secular**	**Arab**
Vaccination coverage (ν)	72.1%	78.1%	63.6%	72.3%	63.7%	88.0%
Comprehended campaign as prosocial (α)	54.8%	48.4%	56.2%	63.6%	66.8%	25.5%
Estimated aware that are prosocial (ρ) ^{b}	69.7%	74.5%	61.6%	71.1%	63.1%	80.2%
Minimum strength of prosocial behavior (κ)	0.30	0.33	0.25	0.30	0.25	0.39
Estimate strength of prosocial behavior $(\kappa)^c$	0.67	0.72	0.59	0.67	0.59	0.83
Sensitivity to infection (γ)	0.095	0.107	0.081	0.096	0.081	0.127

a We assumed a vaccine efficacy of 63%, 94% of the population is eligible for vaccination, the perceived basic reproductive number to be 2.24, the relative risk for an unaware individuals to be approximately 2.14×10^{-4}, the relative risk for an aware individual to be 0.001.

b The percentage of the eligible population that is prosocial was estimated based on the assumption that unaware individuals were approximately 1.22 times more likely to vaccinate than an aware individual (SI)

c Estimated using equation S24

Table S11: The degree of prosociality during the 2013 OPV vaccination campaign estimated from the game theory model and results from the survey under the estimated probability of infection $(1 - S_\infty/(1 - \varepsilon\nu)^a$.

	**National**	**Orthodox**	**Religious**	**Traditional**	**Secular**	**Arab**
Vaccination coverage (ν)	72.1%	78.1%	63.6%	72.3%	63.7%	88.0%
Comprehended campaign as prosocial (α)	54.8%	48.4%	56.2%	63.6%	66.8%	25.5%
Estimated aware that are prosocial (ρ) ^{b}	69.7%	74.5%	61.6%	71.1%	63.1%	80.2%
Minimum strength of prosocial behavior (κ)	6.63 ×10−4	6.63 ×10−4	6.63 ×10−4	6.62 ×10−4	6.64 ×10−4	6.62 ×10−4
Estimate strength of prosocial behavior $(\kappa)^c$	0.33	0.34	0.32	0.33	0.32	0.37
Perceived reproductive number (R_0^P)	1.83	1.97	1.67	1.84	1.67	2.24

a We assumed a vaccine efficacy of 63%, 94% of the population is eligible for vaccination, the perceived basic reproductive number to be 2.24, the relative risk for an unaware individuals to be approximately 2.14×10^{-4}, the relative risk for an aware individual to be 0.001.

b The percentage of the eligible population that is prosocial was estimated based on the assumption that unaware individuals were approximately 1.22 times more likely to vaccinate than an aware individual (SI)

c Estimated using equation S24

Table S12: The degree of prosociality during the 2013 OPV campaign as estimated from the survey data and our approximation from the game theory model.[a]

	Prosocial in population	Approximation ($\alpha\rho\omega$)	Prosocial among aware	Approximation ($\rho\omega$)
National	35.9%	35.88%	65.47%	65.47%
Orthodox	37.5%	33.88%	77.42%	70.01%
Religious	33.1%	32.55%	58.82%	57.92%
Traditional	43.0%	42.52%	67.53%	66.85%
Secular	38.8%	39.61%	58.14 %	59.29%
Arab	21.7%	19.23%	85.11%	75.41%

a We assumed 94% of the population was eligible for vaccination, i.e. $\omega = 0.94$. The approximation is based on the assumption that unaware individuals were approximately 1.22 times more likely to vaccinate than an aware individual (SI).

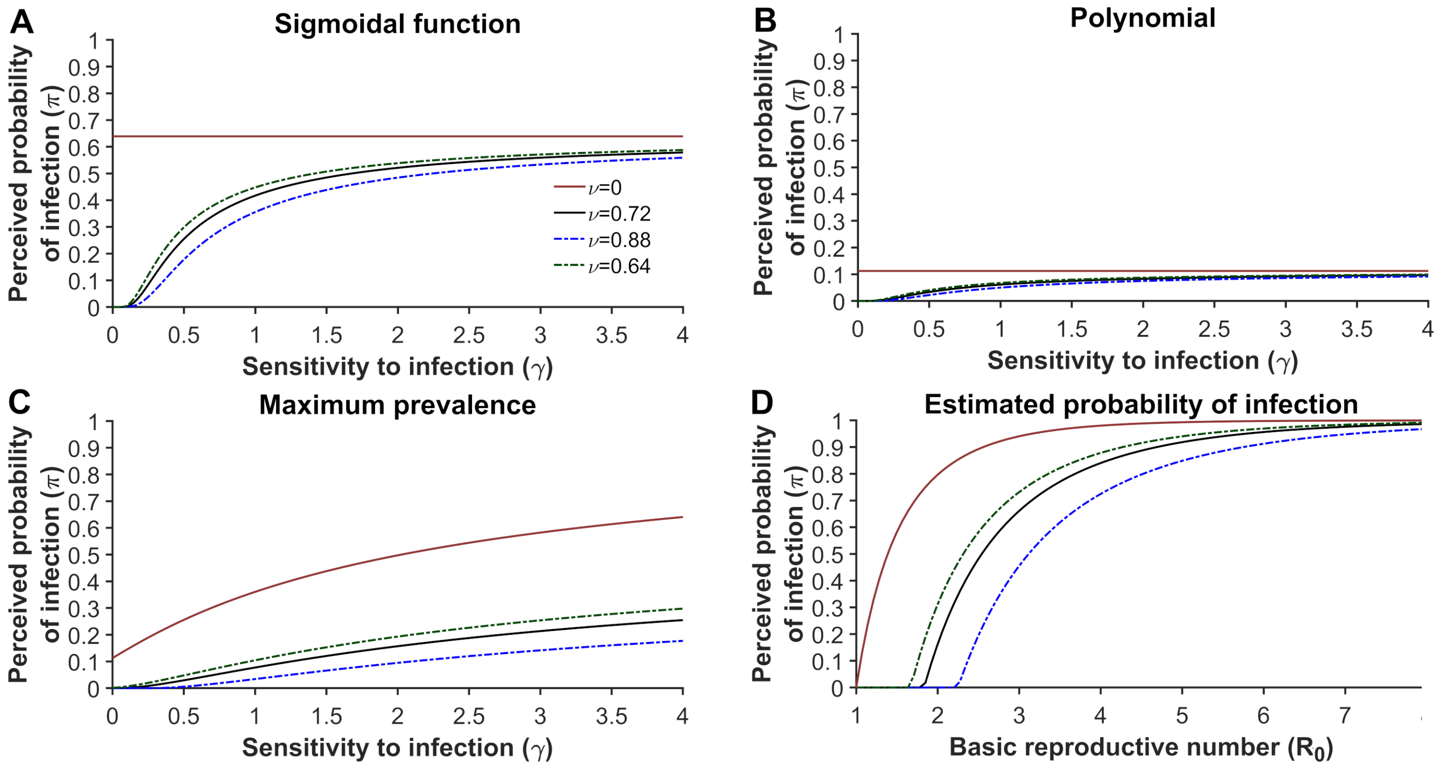

Figure S2: The four perceived probability of infections. The probability of infection using A) the sigmoidal function ($\frac{R_0(1-\varepsilon\nu)^{1/\gamma}}{R_0+(1-\varepsilon\nu)^{1/\gamma}}$), B) the polynomial function ($I_{\max}(R_0,0)(1-\varepsilon\nu)^{1/\gamma}$), C) the maximum prevalence function ($I_{\max}(R_0(1+\gamma),\varepsilon\nu)$ for various sensitivities to infection (γ) and D) the estimated probability of infection ($1 - S_\infty/(1-\varepsilon\nu)$) for various basic reproductive numbers (R_0)). The figures are based on a vaccination coverage of 72% (black), 88% (blue dashed), and 64% (green dashed) with a vaccine efficacy of 63% and 0% vaccination coverage (red), with R_0=1.77 in A)-C) .

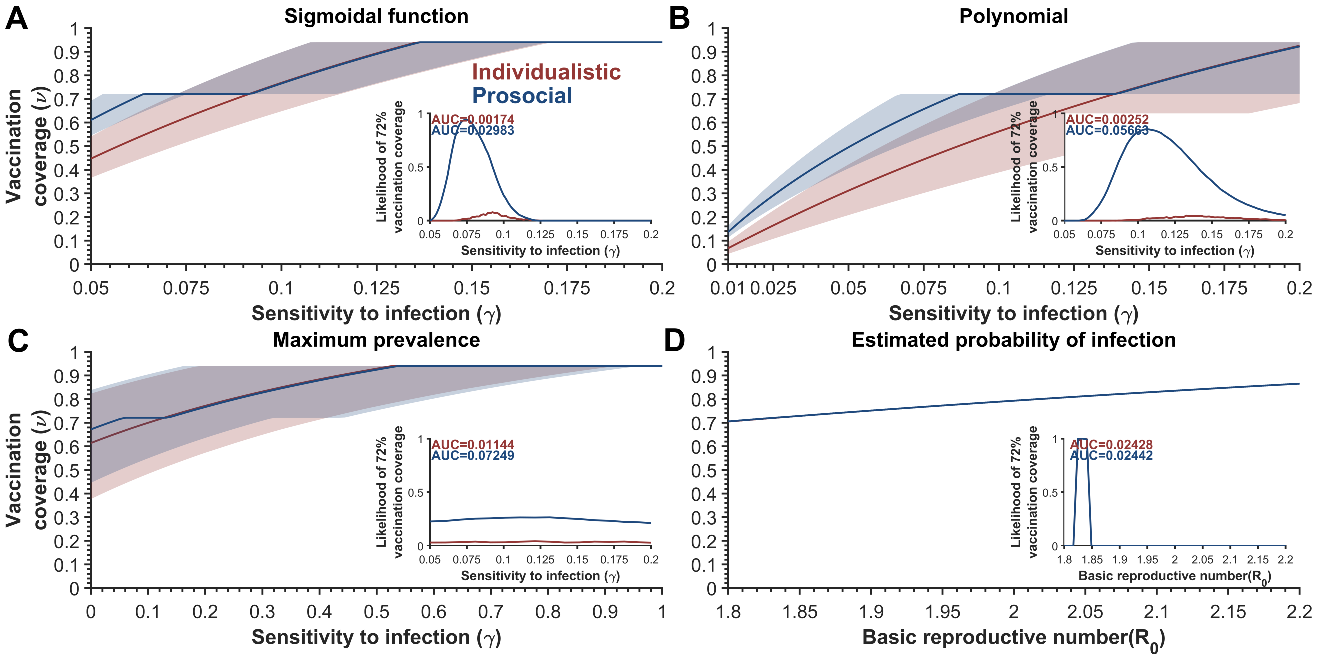

Figure S3: The vaccination coverage predicted by the Nash Equilibrium in an individualistic (red) and prosocial model (blue) for various perceived likelihoods of infection. A) The sigmoidal function $(\frac{R_0(1-\varepsilon\nu)^{1/\gamma}}{R_0+(1-\varepsilon\nu)^{1/\gamma}})$, B) polynomial $(I_{\max}(R_0,0)(1-\varepsilon\nu)^{1/\gamma})$, C) the maximum prevalence function $(I_{\max}(R_0(1+\gamma),\varepsilon\nu)$ for various sensitivities to infection (γ) and D) the estimated probability of infection $(1-S_\infty/(1-\varepsilon\nu))$ for various values of the basic reproductive number (R_0). A)-D) The results are based on 2,500 samples perceived relative risk of paralysis, where R_0 was sampled from its distribution for the sigmoidal function, maximum prevalence, and polynomial functional forms of the perceived likelihoods of infection.

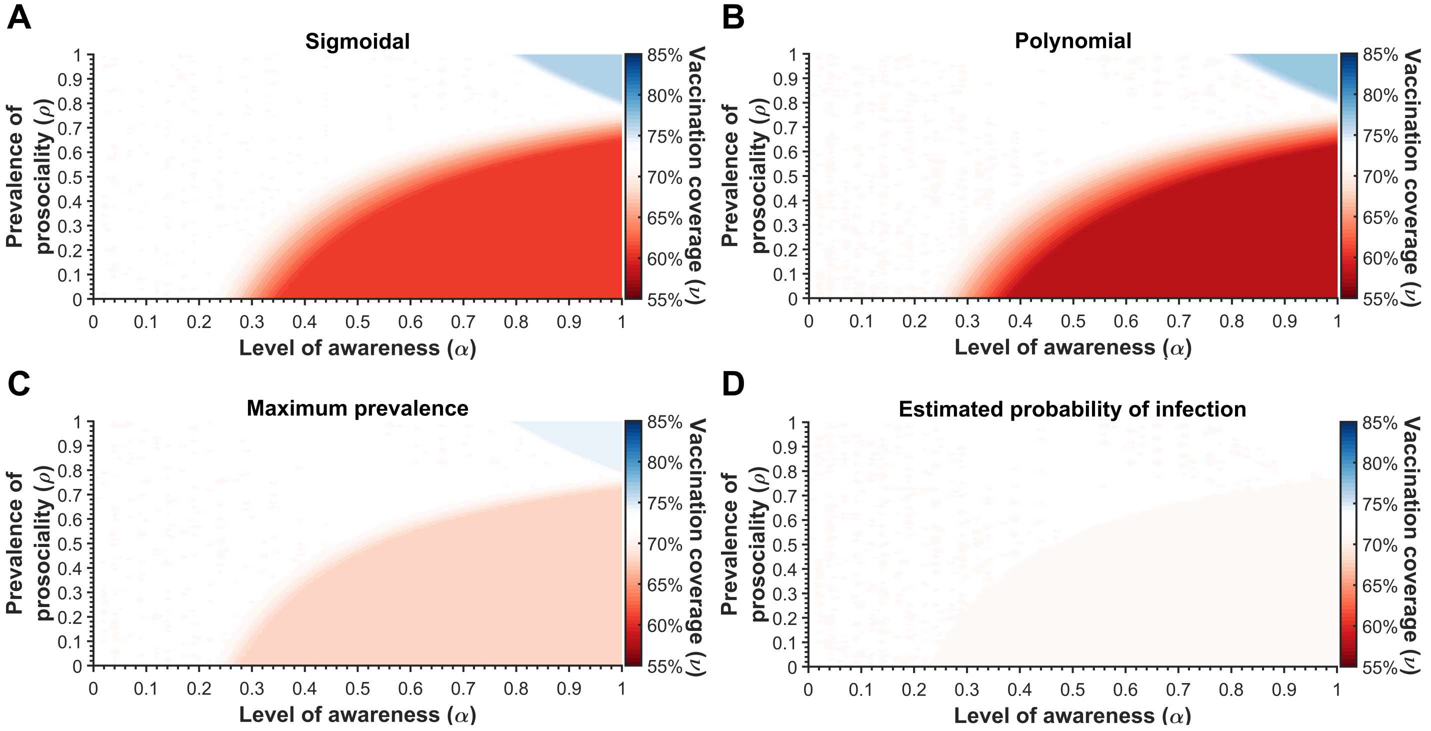

Figure S4: The vaccination coverage predicted by the Nash Equilibrium across varying proportions of aware (α) and prosocial (ρ) individuals in Israel, when unaware individuals update their perceived relative risk. The 72% vaccination coverage is based on A) the sigmoidal function ($\frac{R_0(1-\varepsilon\nu)^{1/\gamma}}{R_0+(1-\varepsilon\nu)^{1/\gamma}}$), B) the polynomial function ($I_{\max}(R_0, 0)(1-\varepsilon\nu)^{1/\gamma}$), C) the maximum prevalence function ($I_{\max}(R_0(1+\gamma), \varepsilon\nu)$ and D) the estimated probability of infection ($1 - S_\infty/(1-\varepsilon\nu)$).The value of the perceived basic reproductive number, the sensitivity to infection, and the strength of prosocial behavior are described in Table1 and Table S9-S11 for each of the functional forms. The relative risk for the aware and unaware and the fraction of the population eligible for vaccination are described in SI. The blue denotes a coverage greater than baseline, red indicates a coverage lower than baseline, and white represents the baseline coverage of 72%

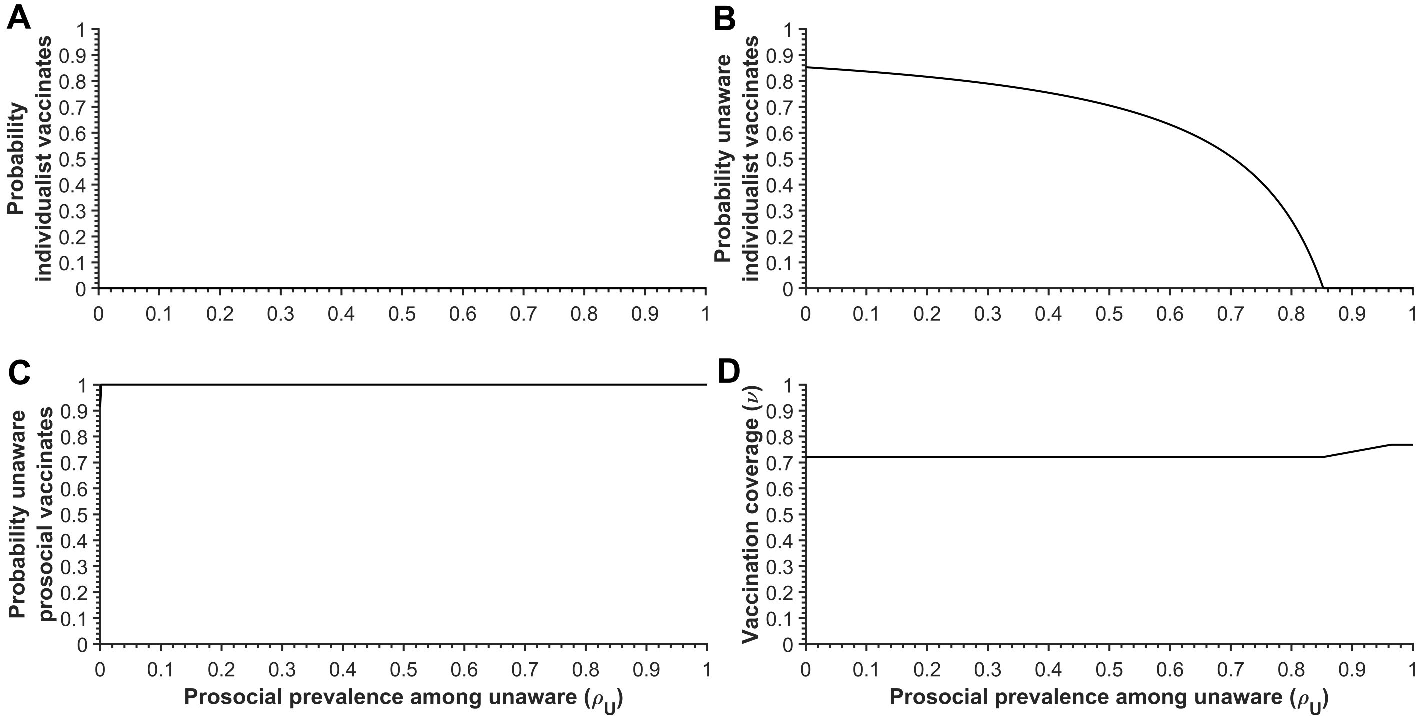

Figure S5: The probability of vaccinating for an individualist, an unaware individual, an unaware prosocial individual, and the vaccination coverage for various levels prosociality among the unaware in Israel when unaware individuals do not update their perceived risk. The probability of vaccinating for A) an individualist, B) an unaware individualist, and C) an unaware prosocial individual. D) The vaccination coverage among the entire population, assuming 94% of the population is eligible for vaccination These results are based on the perceived parameters for Israel described in Table 1 and a 72% vaccination coverage and the conservative assumption that 100% of the unaware population have been informed about the prosocial campaign.

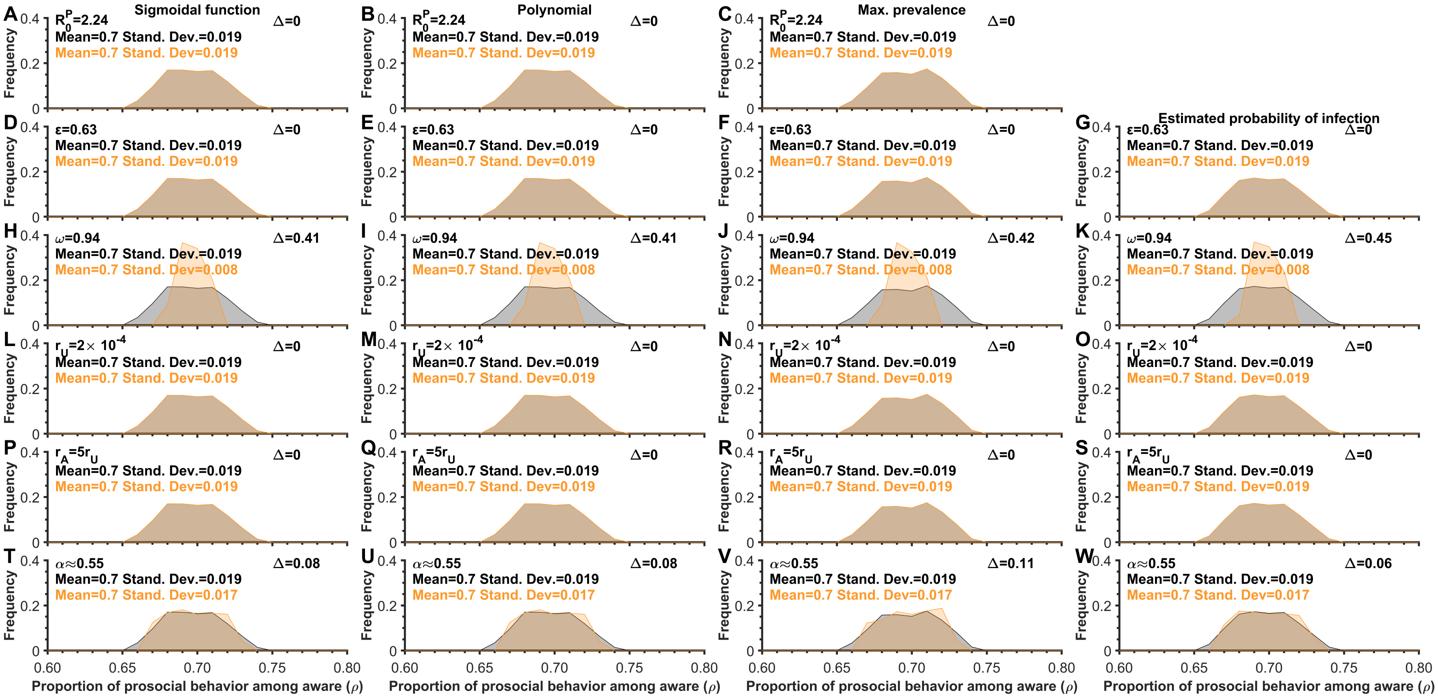

Figure S6: The change in distribution of the estimated proportion of prosocial behavior for various parameters and probabilities of infection. We consider the impact of fixing (orange) the A)-C) the perceived basic reproductive number, D)-G) the vaccine efficacy, H)-K) the proportion of the population eligible for OPV, L)-0) the unaware relative risk, P)-S) difference in relative risk between aware and unaware, and T)-W) the level of comprehension. We consider these effects for the (far left column) the sigmoidal function, (middle left column) the polynomial, (middle right column) maximum prevalence and (far right column) the estimated probability of infection. These results are based on 5,000 independent samples of the parameters from their specified ranges described in Table S8 (black).

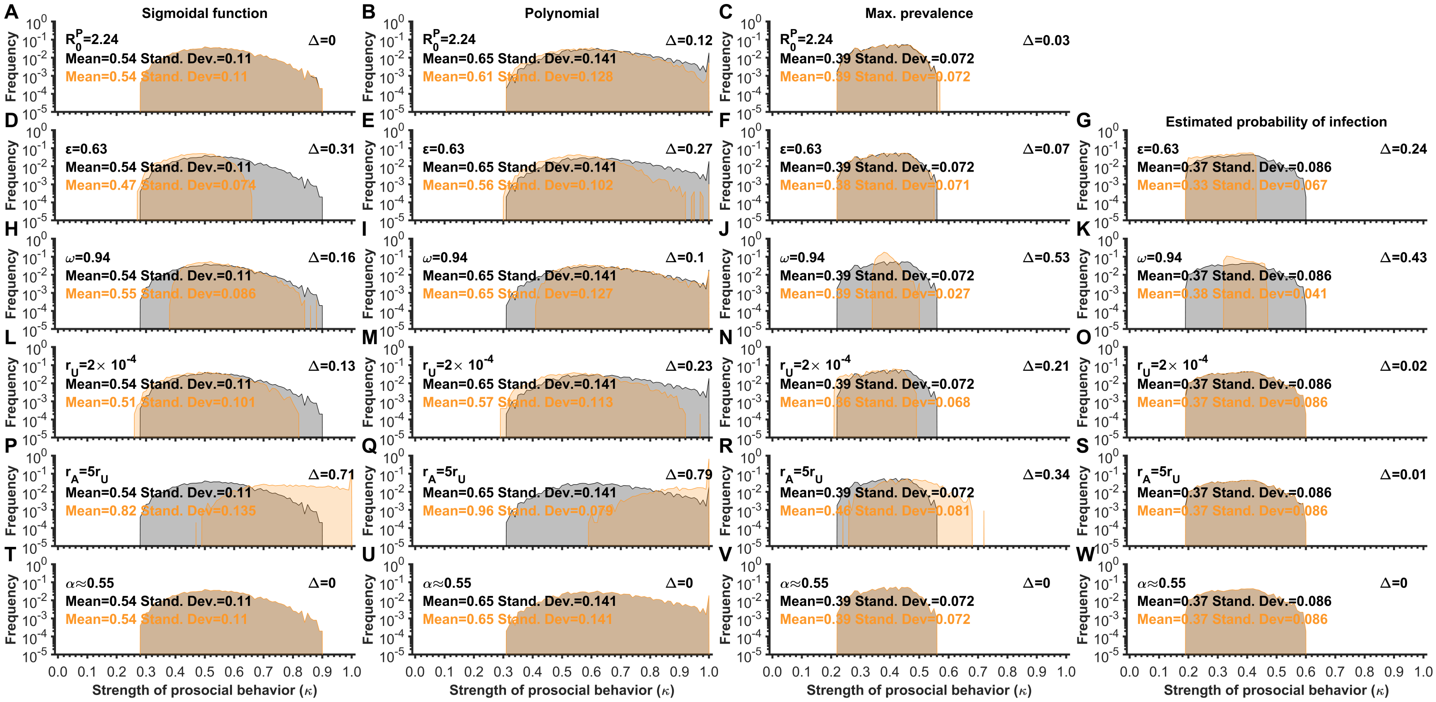

Figure S7: The change in distribution of the estimated strength of prosocial behavior for various parameters and probabilities of infection. We consider the impact of fixing (orange) the A)-C) the perceived basic reproductive number, D)-G) the vaccine efficacy, H)-K) the proportion of the population eligible for OPV, L)-0) the unaware relative risk, P)-S) difference in relative risk between aware and unaware, and T)-W) the level of comprehension. We consider these effects for the (far left column) the sigmoidal function, (middle left column)the polynomial, (middle right column) maximum prevalence and (far right column) the estimated probability of infection. These results are based on 5,000 independent samples of the parameters from their specified ranges described in Table S8 (black).

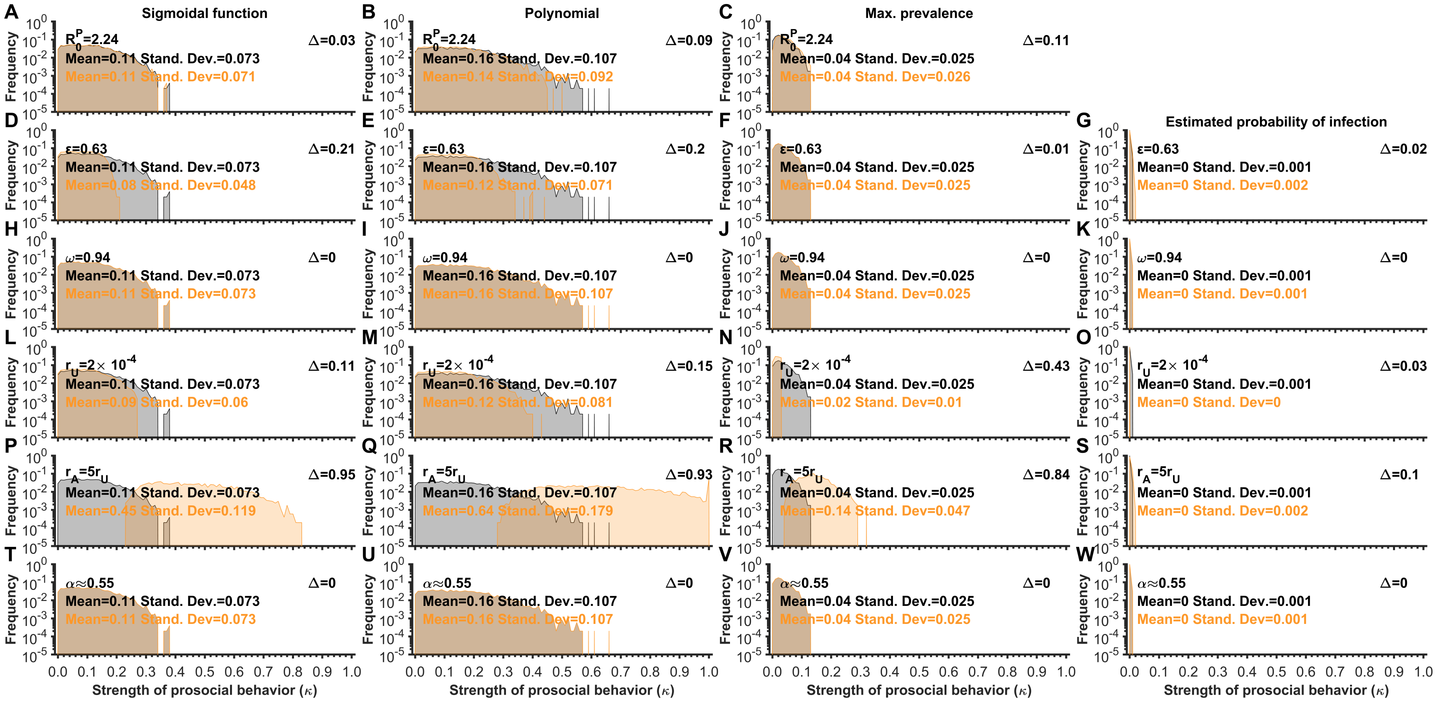

Figure S8: The change in distribution of the minimum strength of prosocial behavior for various parameters and probabilities of infection. We consider the impact of fixing (orange) the A)-C) the perceived basic reproductive number, D)-G) the vaccine efficacy, H)-K) the proportion of the population eligible for OPV, L)-0) the unaware relative risk, P)-S) difference in relative risk between aware and unaware, and T)-W) the level of comprehension. We consider these effects for the We consider these effects for the (far left column) the sigmoidal function, (middle left column) the polynomial, (middle right column) maximum prevalence and (far right column) the estimated probability of infection. These results are based on 5,000 independent samples of the parameters from their specified ranges described in Table S8 (black).

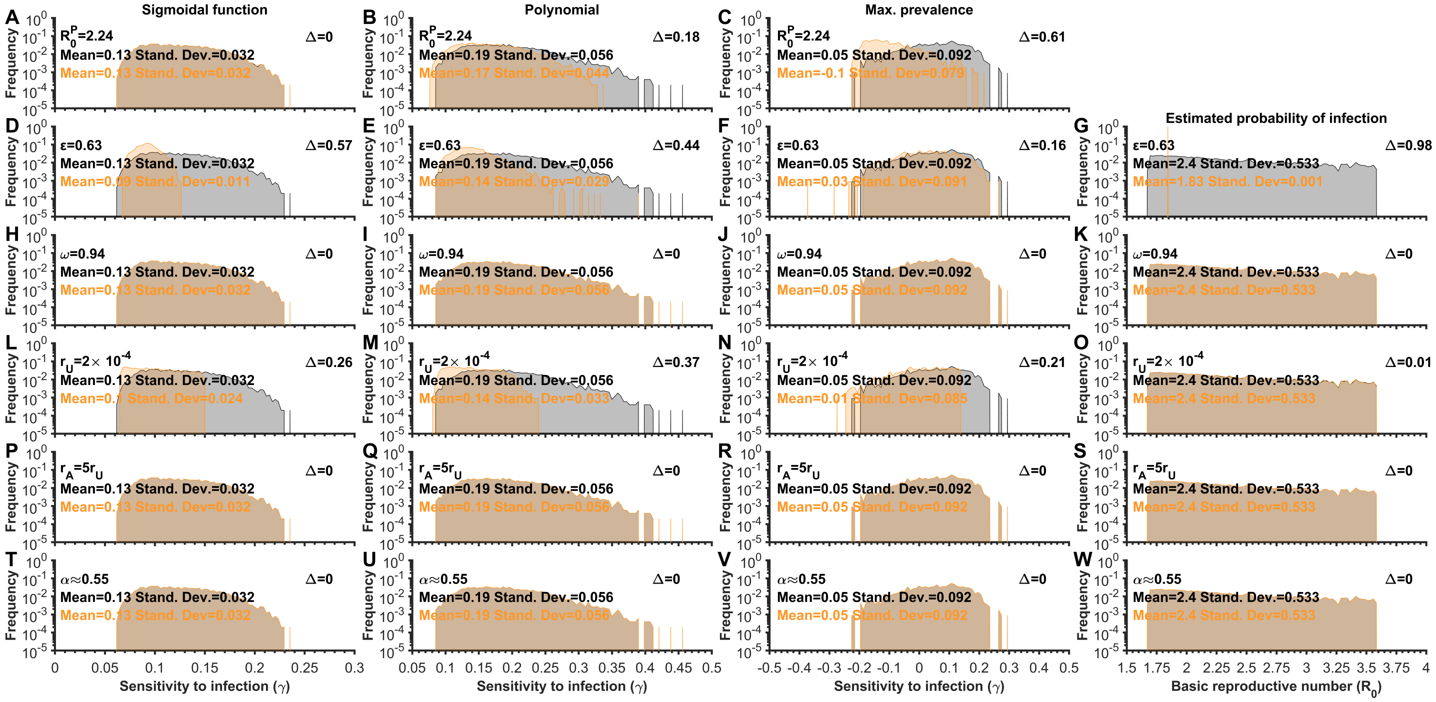

Figure S9: The change in distribution of the estimated sensitivity to infection for various parameters and probabilities of infection. We consider the impact of fixing (orange) the A)-C) the perceived basic reproductive number, D)-G) the vaccine efficacy, H)-K) the proportion of the population eligible for OPV, L)-0) the unaware relative risk, P)-S) difference in relative risk between aware and unaware, and T)-W) the level of comprehension. We consider these effects for the (far left column) the sigmoidal function, (middle left column)the polynomial, (middle right column) maximum prevalence and (far right column) the estimated probability of infection. These results are based on 5,000 independent samples of the parameters from their specified ranges described in Table S8 (black).

References

[1] B Velan, G Kaplan, and A Huppert. Analysis of the public response to live oral polio vaccination during the 2013 silent poliovirus outbreak in israel. report to the "the israeli national institute for". *Health policy research*, 2015.

[2] Dale J Cohen and Katherine E Bruce. Sex and mortality: Real risk and perceived vulnerability. *J. Sex Res.*, 34(3):279–291, 1997.

[3] Alison P Galvani, Timothy C Reluga, and Gretchen B Chapman. Long-standing influenza vaccination policy is in accord with individual self-interest but not with the utilitarian optimum. *Proc. Natl. Acad. Sci. U. S. A.*, 104(13):5692–5697, 27 March 2007.

[4] Eunha Shim, Gretchen B Chapman, Jeffrey P Townsend, and Alison P Galvani. The influence of altruism on influenza vaccination decisions. *J. R. Soc. Interface*, 9(74):2234–2243, 7 September 2012.

[5] Joel C Miller. A note on the derivation of epidemic final sizes. *Bulletin of mathematical biology*, 74(9):2125–2141, 2012.

[6] James D Murray. *Mathematical Biology: I. An Introduction.* Springer Science & Business Media, 2 February 2011.

[7] J Sawyer. The altruism scale: a measure of co-operative, individualistic, and competitive interpersonal orientation. *AJS*, 71(4):407–416, January 1966.

[8] Asif Raza Khowaja, Sher Ali Khan, Naveeda Nizam, Saad Bin Omer, and Anita Zaidi. Parental perceptions surrounding polio and self-reported non-participation in polio supplementary immunization activities in karachi, pakistan: a mixed methods study. *Bull. World Health Organ.*, 90(11):822–830, 1 November 2012.

[9] Gillian K SteelFisher, Robert J Blendon, Sherine Guirguis, Amanda Brulé, Narayani Lasala-Blanco, Michael Coleman, Vincent Petit, Mashrur Ahmed, Noah Mataruse, Melissa Corkum, Mazhar Nisar, Eran N Ben-Porath, Susan Gigli, and Christoph Sahm. Threats to polio eradication in high-conflict areas in pakistan and nigeria: a polling study of caregivers of children younger than 5 years. *Lancet Infect. Dis.*, 15(10):1183–1192, October 2015.

[10] Joshua A Salomon, Juanita A Haagsma, Adrian Davis, Charline Maertens de Noordhout, Suzanne Polinder, Arie H Havelaar, Alessandro Cassini, Brecht Devleesschauwer, Mirjam Kretzschmar, Niko Speybroeck, Christopher J L Murray, and Theo Vos. Disability weights for the global burden of disease 2013 study. *Lancet Glob Health*, 3(11):e712–23, November 2015.

[11] World Health Organization. *The Global Burden of Disease: 2004 Update.* World Health Organization, 2008.

[12] Robert Böhm, Cornelia Betsch, and Lars Korn. Selfish-rational non-vaccination: Experimental evidence from an interactive vaccination game. *J. Econ. Behav. Organ.*

[13] Mark Spranca, Elisa Minsk, and Jonathan Baron. Omission and commission in judgment and choice. *J. Exp. Soc. Psychol.*, 27(1):76–105, 1991.

[14] D A Asch, J Baron, J C Hershey, H Kunreuther, J Meszaros, I Ritov, and M Spranca. Omission bias and pertussis vaccination. *Med. Decis. Making*, 14(2):118–123, April 1994.

[15] Emmanuel Vidor and Stanley A Plotkin. Poliovirus vaccine-inactivated. *Vaccines. 6th edition. Plotkin SA, Orenstein WA, Offit PA, editores. Elsevier*, 2013.

[16] L H de Oliveira and C J Struchiner. Vaccine-associated paralytic poliomyelitis: a retrospective cohort study of acute flaccid paralyses in brazil. *Int. J. Epidemiol.*, 29(4):757–763, August 2000.

[17] WHO — poliomyelitis. 22 April 2016.

[18] The disease and vaccine, ministry of health. `http://www.health.gov.il/English/Topics/Vaccination/two_drops/Pages/Vaccination.aspx`. Accessed: 2016-5-13.

[19] Rami Yaari, Yaari Rami, for the POG group, Kaliner Ehud, Grotto Itamar, Katriel Guy, Moran-Gilad Jacob, Sofer Danit, Mendelson Ella, Miller Elizabeth, Huppert Amit, E Anis, E Kopel, Y Manor, O Mor, L Shulman, R Singer, and M Weil. Modeling the spread of polio in an IPV-vaccinated population: lessons learned from the 2013 silent outbreak in southern israel. *BMC Med.*, 14(1), 2016.

[20] Matthew R Behrend, Hao Hu, Karima R Nigmatulina, and Philip Eckhoff. A quantitative survey of the literature on poliovirus infection and immunity. *Int. J. Infect. Dis.*, 18:4–13, January 2014.

[21] WHO — World Health Organization. WHO — immunization country profile.
